# Linear ubiquitination is involved in the pathogenesis of optineurin-associated amyotrophic lateral sclerosis

**DOI:** 10.1038/ncomms12547

**Published:** 2016-08-24

**Authors:** Seshiru Nakazawa, Daisuke Oikawa, Ryohei Ishii, Takashi Ayaki, Hirotaka Takahashi, Hiroyuki Takeda, Ryuichiro Ishitani, Kiyoko Kamei, Izumi Takeyoshi, Hideshi Kawakami, Kazuhiro Iwai, Izuho Hatada, Tatsuya Sawasaki, Hidefumi Ito, Osamu Nureki, Fuminori Tokunaga

**Affiliations:** 1 Laboratory of Molecular Cell Biology, Institute for Molecular and Cellular Regulation, Gunma University, 3-39-15 Showa-machi, Maebashi, Gunma 371-8512, Japan; 2 Department of Thoracic and Visceral Organ Surgery, Gunma University Graduate School of Medicine, 3-39-22 Showa-machi, Maebashi, Gunma 371-8511, Japan; 3 Department of Pathobiochemistry, Graduate School of Medicine, Osaka City University, 1-4-3 Asahi-machi, Abeno-ku, Osaka 545-8585, Japan; 4 Department of Biological Sciences, Graduate School of Science, The University of Tokyo, 2-11-16 Yayoi, Bunkyo-ku, Tokyo 113-0032, Japan; 5 Department of Neurology, Wakayama Medical University, 811-1, Kimiidera, Wakayama, Wakayama 641-8510, Japan; 6 Department of Neurology, Kyoto University Graduate School of Medicine, Sakyo-ku, Shogoin, Kyoto 606-8507, Japan; 7 Proteo-Science Center, Ehime University, Matsuyama, Ehime 790-8577, Japan; 8 Department of Epidemiology, Research Institute for Radiation Biology and Medicine, Hiroshima University, 1-2-3 Kasumi, Minami-ku, Hiroshima 734-8553, Japan; 9 Department of Molecular and Cellular Physiology, Graduate School of Medicine, Kyoto University, Yoshida-konoe-cho, Sakyo-ku, Kyoto 606-8501, Japan; 10 Laboratory of Genome Science, Biosignal Genome Resource Center, Institute for Molecular and Cellular Regulation, Gunma University, 3-39-15 Showa-machi, Maebashi, Gunma 371-8512, Japan

## Abstract

Optineurin (*OPTN)* mutations cause neurodegenerative diseases, including amyotrophic lateral sclerosis (ALS) and glaucoma. Although the ALS-associated E478G mutation in the UBAN domain of OPTN reportedly abolishes its NF-κB suppressive activity, the precise molecular basis in ALS pathogenesis still remains unclear. Here we report that the OPTN-UBAN domain is crucial for NF-κB suppression. Our crystal structure analysis reveals that OPTN-UBAN binds linear ubiquitin with homology to NEMO. TNF-α-mediated NF-κB activation is enhanced in *OPTN*-knockout cells, through increased ubiquitination and association of TNF receptor (TNFR) complex I components. Furthermore, OPTN binds caspase 8, and OPTN deficiency accelerates TNF-α-induced apoptosis by enhancing complex II formation. Immunohistochemical analyses of motor neurons from OPTN-associated ALS patients reveal that linear ubiquitin and activated NF-κB are partially co-localized with cytoplasmic inclusions, and that activation of caspases is elevated. Taken together, OPTN regulates both NF-κB activation and apoptosis via linear ubiquitin binding, and the loss of this ability may lead to ALS.

## Abstract (web summary)

Mutations in optineurin are associated with neurodegenerative diseases, including amyotrophic lateral sclerosis. Here, the authors report the structure of the ubiquitin binding domain of optineurin, which binds linear ubiquitin with homology to NEMO, and explore the function of this domain.

## Graphical abstract

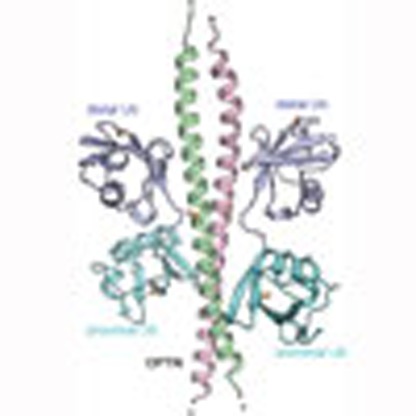

The nuclear factor-κB (NF-κB) pathway is a central signal transduction pathway regulating inflammation, adaptive and innate immune responses, and cell death, mostly through transcriptional targets. Therefore, impaired NF-κB activity is implicated in diverse disorders, including inflammatory, autoimmune and neurodegenerative diseases, metabolic syndrome and cancers^1^,^2^. Posttranslational modifications, such as phosphorylation and ubiquitination, regulate the NF-κB activation pathways^3^. In ubiquitination, a Lys63 (K63)-linked polyubiquitin chain, generated by tumour-necrosis factor (TNF) receptor-associated factor proteins, plays a crucial role as a scaffold to recruit the TAK1 (transforming growth factor-β-activated kinase 1)–TAK1-binding protein complex, which then phosphorylates inhibitor of NF-κB (IκB) kinase β (IKKβ)^4^. IKK, composed of two kinase subunits (IKKα and IKKβ) and a non-catalytic regulatory subunit, NEMO, phosphorylates IκB. Phosphorylated IκB is degraded through the ubiquitin–proteasome system, allowing the intranuclear translocation of NF-κB and subsequent induction of transcriptional targets.

In parallel to NF-κB activation mediated by the K63-linked ubiquitin chain, we previously showed that the linear ubiquitin chain assembly complex (LUBAC) composed of HOIL-1L (also known as RBCK1), HOIP (RNF31) and SHARPIN, specifically generates the amino-terminal Met1 (M1)-linked linear polyubiquitin chain^5^. NF-κB activation is induced by linear ubiquitination of NEMO and RIP1 by LUBAC, on stimulation by proinflammatory cytokines^6^,^7^. The linear ubiquitin chain then functions as a scaffold to recruit the IKK complex via the linear ubiquitin-specific binding site of NEMO, named UBAN domain^6^,^8^,^9^. IKK complexes recruited to linear ubiquitin chains are then activated by *trans*-phosphorylation, which finally initiates canonical NF-κB signalling by subsequent phosphorylation of IκB^10^. Thus, proteins with linear ubiquitin-specific binding domains, such as the UBAN domain, play pivotal roles in the recognition and regulation of linear ubiquitin-mediated signalling.

Optineurin (OPTN, also called FIP2 and NRP) and A20-binding IκB proteins contain a UBAN domain^11^. Among them, OPTN exhibits the highest sequence homology to NEMO^12^. Unlike NEMO, OPTN does not interact with IKKα and IKKβ^13^; however, OPTN suppresses NF-κB activity by competing with NEMO function^14^. Furthermore, OPTN binds IKK subfamily protein kinases, such as TANK-binding kinase 1 (TBK1) and IKKɛ (also called IKKi), thus contributing to virus-triggered interferon IFN production pathways^15^,^16^ and *Salmonella*-induced autophagy^17^. Recently, OPTN, as well as NDP52, was identified as major factors in mitophagy and OPTN phosphorylation by TBK1 regulates these process^18^,^19^,^20^. OPTN also reportedly associates with Rab8 and myosin VI, to regulate vesicular trafficking and Golgi morphology^21^,^22^. Importantly, mutations in *OPTN* are associated with neurodegenerative disorders. First, mutations in *OPTN*, such as E50K, were identified as inducing primary open-angle glaucoma (POAG)^23^. Subsequently, other mutations in *OPTN*, such as Q398X and E478G, were detected in patients with familial amyotrophic lateral sclerosis (ALS)^24^. ALS is a progressive neurodegenerative disease affecting motor neurons and, although most cases are sporadic, about 10% are familial. Currently, mutations in ∼20 genes, such as *superoxide dismutase 1* (*SOD1*), *TAR DNA-binding protein 43* (*TDP-43*), *FUS*, *ubiquilin 2*, *p62* (*SQSTM1*), *p97* (*VCP*), *TBK1* and *OPTN*, were identified as responsible for familial ALS. Mutations in *OPTN* are minor, but evident for familial ALS^25^,^26^. Moreover, variants of *OPTN* are also linked with Paget's disease of the bone^27^ and frontotemporal lobe dementia^28^. However, the roles of OPTN in the pathophysiology of neurodegenerative disorders have yet to be determined.

Here we show that among the *OPTN* mutations reported in POAG and ALS, most of the ALS-associated mutants fail to suppress NF-κB activation. OPTN mutants without inhibitory effects have either a mutation or deletion of the UBAN domain. The crystal structure of OPTN-UBAN in complex with linear tetraubiquitin reveals that the residues involved in linear ubiquitin binding correspond to the residues crucial for suppression of NF-κB activation. Furthermore, we analyse the NF-κB activation by constructing CRISPR/Cas9-directed *OPTN*-knockout (KO) cells. We find that OPTN also functions in the TNF-α-induced extrinsic apoptosis pathway. By examining *OPTN*-associated ALS patients' samples, we detect linear ubiquitin and activated NF-κB in cytoplasmic inclusions, with association of enhanced apoptosis. Taken together, our findings indicate that OPTN is involved in critical signalling pathways and the disruption of these cellular functions of OPTN is involved in ALS pathogenesis.

## Results

### Dysregulation of NF-κB by ALS-associated *OPTN* mutations

**Figure 1:**
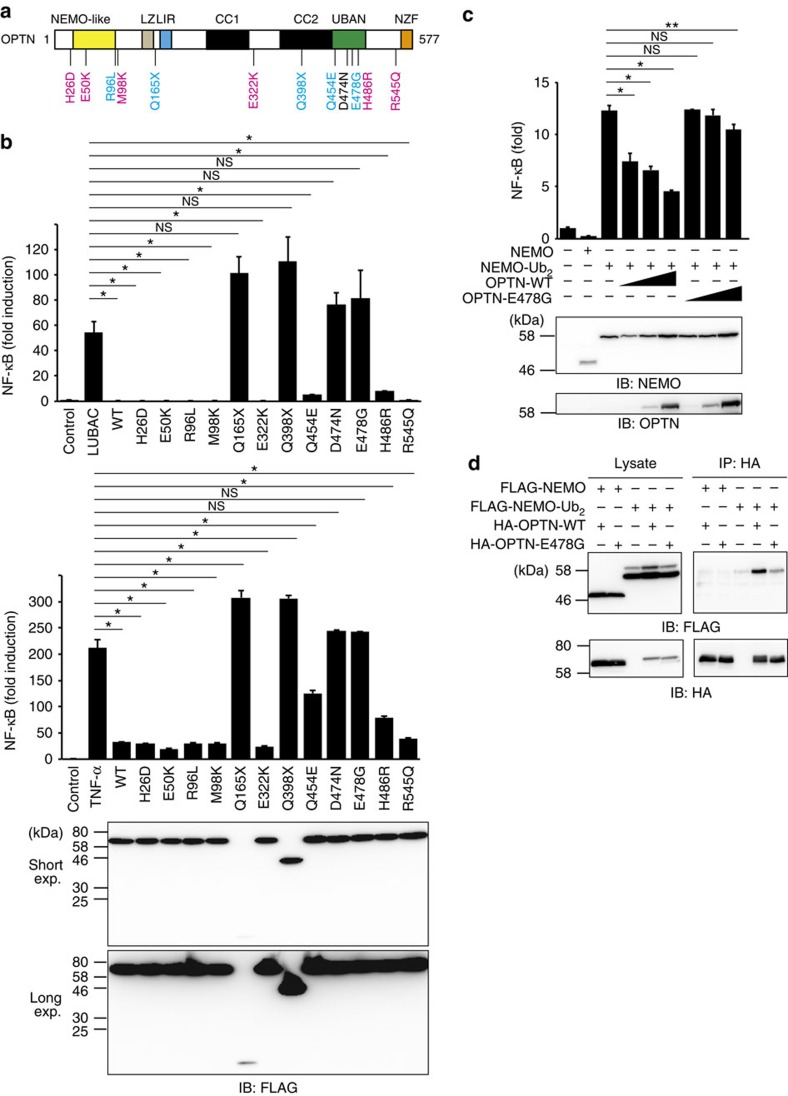
ALS-associated OPTN mutants fail to suppress NF-κB activity. (**a**) Domain structure of OPTN and disease-associated mutations. CC, coiled-coil; LZ, leucine zipper; LIR, LC3-interacting region; NZF, Npl4-type zinc finger; UBAN, ubiquitin binding in A20-binding IκB (ABIN) and NEMO proteins. Blue, ALS-associated mutations; pink, POAG-associated mutations. (**b**) Effects of WT and mutants of OPTN on LUBAC- and TNF-α-induced NF-κB activation were examined by luciferase assays in HEK293T cells. Expression of WT and mutants of FLAG-OPTNs is shown by immunoblotting. (**c**) Effects of WT and E478G mutant of OPTN on linear diubiquitin-conjugated NEMO were examined as in **b**. (**b**,**c**) Induction folds of NF-κB activity by luciferase assay are shown as mean±s.e.m. (*n*=3). Statistical significance was assessed using one-way analysis of variance. *P<0.001 and **P<0.01, NS, not significant. (**d**) WT-OPTN, but not the E478G mutant, binds to linear di-ubiquitinated NEMO. Immunoprecipitation and immunoblotting were performed as indicated.

OPTN consists of multiple domains, such as leucine zipper, LC3-interacting region (LIR), two coiled-coil (CC1 and CC2), UBAN and Npl4-type zinc finger (Fig. 1a)^12^. *In vitro* experiments have linked OPTN to various signalling pathways. However, the domains and pathways involved in the pathogenesis of OPTN-associated diseases still remain unclear. At present, missense mutations of *OPTN*, such as H26D, E50K, M98K, E322K, H486R and R545Q, were linked to POAG, and R96L, Q165X, Q398X, Q454E and E478G (X denotes a stop codon) mutations were identified from familial ALS patients^12^,^29^ (Fig. 1a). Furthermore, we employed the non-pathogenic D474N mutant, with a mutation in the UBAN domain, as its abilities to induce defects in endocytic trafficking, NF-κB and IFN signalling, and mitophagy have been well characterized^14^,^19^,^30^. To identify the residues and domains directly involved in the pathogenesis of ALS and POAG, we performed a comprehensive analysis by using these 12 OPTN mutants. As OPTN shows high similarity with NEMO, a crucial factor in the NF-κB activation pathway, we examined the effect of these OPTN mutants on LUBAC- and TNF-α-mediated NF-κB activation by luciferase reporter assays (Fig. 1b). Expression of OPTN wild type (WT) or POAG-associated OPTN mutants strongly suppressed LUBAC- and TNF-α-mediated NF-κB activation. However, the inhibitory effect was lost in the Q165X, Q398X, D474N and E478G mutants, and the Q454E and H486R mutants also showed partially reduced inhibitory effects. Although it was previously reported that missense mutations in the UBAN domain of OPTN, such as D474N and E478G, abolished the suppressive effect of OPTN on TNF-α-mediated NF-κB activation^14^,^24^, we confirmed here that the UBAN domain is also critical for LUBAC-mediated NF-κB regulation. Interestingly, most of the OPTN mutants that lost the inhibitory effect were those with ALS-associated mutations, suggesting that impaired suppression of NF-κB activation due to mutations in *OPTN* may play a key role in the pathogenesis of OPTN-associated ALS.

Next, to identify the inhibitory target of OPTN, we examined the effects of OPTN-WT and OPTN-E478G mutant on NF-κB activation induced by overexpression of NF-κB activators (Supplementary Fig. 1). OPTN reportedly suppressed NF-κB activation induced by RIP1, but not that by a constitutively active mutant of IKKβ (IKKβ-EE)^14^. In addition, OPTN lacked inhibitory effects on the non-canonical NF-κB activation pathway induced by NF-κB-inducing kinase. The E478G mutant could not inhibit NF-κB activation in either case. We also analysed the effect of OPTN on NF-κB activation induced by linearly di-ubiquitinated NEMO (Fig. 1c)^31^. OPTN-WT, but not the E478G mutant, suppressed NF-κB activated by di-ubiquitinated NEMO. This correlates with the ability of OPTN to bind NEMO, as OPTN-WT could bind linearly di-ubiquitinated NEMO, whereas the E478G mutant had drastically reduced binding ability (Fig. 1d). These results suggested that OPTN suppresses the canonical IKK activation process and linear ubiquitin chain binding by OPTN-UBAN is necessary for this effect.

In contrast to IKKα and IKKβ, which do not bind to OPTN, TBK1 and IKKɛ bound not only OPTN-WT but also OPTN mutants, such as E478G and Q398X. Furthermore, endogenous OPTN and TBK1 were constitutively associated during TNF-α stimulation (Supplementary Fig. 2a–c). Moreover, the NF-κB and IFN pathways activated by TBK1 were not suppressed by OPTN-WT or E478G (Supplementary Fig. 2d,e), collectively indicating that the UBAN domain is not involved in the collaborative OPTN functions with TBK1/IKKɛ.

### UBAN domain of OPTN selectively binds to linear ubiquitin

**Figure 2:**
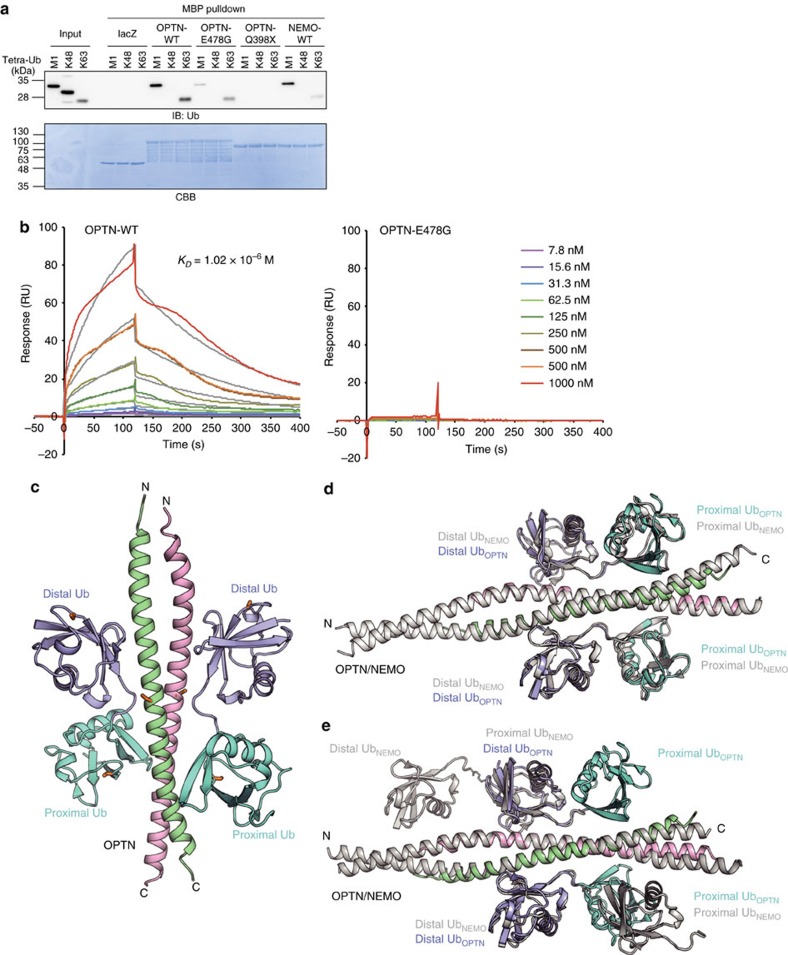
OPTN-UBAN domain is critical for linear ubiquitin binding. (**a**) The E478G mutation abrogated linear ubiquitin binding. *In vitro* MBP pull-down experiments using linear (M1)-, K48- or K63-linked tetraubiquitins and MBP-fused lacZ, OPTN-WT, E478G, Q398X and NEMO-WT were performed, and the bound ubiquitin chain was detected by immunoblotting. (**b**) Kinetic analyses of OPTN and linear ubiquitin. Linear tetraubiquitin was immobilized and various concentrations of OPTN-WT or E478G were tested. Grey lines, a global fit to a 1:1 interaction model. (**c**) Crystal structure of OPTN-UBAN in complex with linear diubiquitin (crystallized in the presence of linear tetraubiquitin). Each subunit of OPTN is coloured green and pink, respectively. The distal and proximal ubiquitin moieties are coloured purple and cyan, respectively. Phosphorylation sites, such as Ser65 in ubiquitin and Ser473 in OPTN, are coloured orange. (**d**) Superimposition of the structures of OPTN·linear diubiquitin and NEMO·linear diubiquitin (grey) (PDB 2ZVN). (**e**) Superimposition of the structures of OPTN·linear diubiquitin and NEMO·K63 diubiquitin (grey) (PDB 3JSV).

We next examined the ubiquitin-binding features of OPTN by a maltose-binding protein (MBP) pull-down analysis (Fig. 2a). OPTN-WT efficiently bound linear (M1)- and K63-linked tetraubiquitin, but not K48-linked tetraubiquitin. The E478G mutation drastically reduced OPTN binding to linear ubiquitin, suggesting that the E478 residue in the UBAN domain is critical for linear ubiquitin binding. OPTN-Q398X did not bind to either type of ubiquitin and NEMO-WT efficiently bound to linear ubiquitin^9^,^32^. A surface plasmon resonance (SPR) analysis revealed that the association and dissociation rates of OPTN-WT and linear tetraubiquitin were 5.09 × 10^3^ M^–1^ s^–1^ and 5.19 × 10^−3^ s^–1^, respectively, and the resultant affinity (*K*_D_) was 1.02 × 10^−6^ M (Fig. 2b). However, OPTN-E478G did not bind to linear ubiquitin in the SPR analysis.

To clarify the molecular mechanism of linear ubiquitin recognition by OPTN, we crystallized the human OPTN CC2-UBAN region (residues 416–510) in complex with linear tetraubiquitin and determined the complex structure at 2.7 Å (Fig. 2c). The asymmetric unit contained four OPTN CC2-UBAN monomers, which form two parallel coiled-coil dimer structures, and two tetraubiquitin molecules (Supplementary Fig. 3). Although we used the OPTN CC2-UBAN region for crystallization experiments, the electron density for the majority of the CC2 region in all OPTN molecules was very weak and thus we modelled only the UBAN motif with some extensions on both sides (residues 445–505 for the longest chain). In the crystal, the third and fourth ubiquitin moieties of tetraubiquitin bind to one side of OPTN-UBAN in the same asymmetric unit, whereas the first and second ubiquitin moieties bind to the other side of OPTN-UBAN in the adjacent cell (Supplementary Fig. 3b,c). Thus, there are two OPTN–diubiquitin complexes, composed of one OPTN dimer and two diubiquitins, in the asymmetric unit.

The UBAN domain structure of OPTN·diubiquitin superimposed well on that of NEMO-UBAN·linear diubiquitin, with a root-mean square deviation of 0.72 Å for 62 Cα atoms (Fig. 2d). OPTN-UBAN binds to diubiquitin in a similar manner to that observed in the structure of the NEMO-UBAN·linear diubiquitin complex^9^ rather than that in the NEMO-UBAN·K63 diubiquitin complex (Fig. 2d,e and Supplementary Figs 4 and 5)^33^. The distal ubiquitin is recognized mainly by hydrophobic interactions, with additional polar interactions (Supplementary Fig. 4a). In contrast, the proximal ubiquitin is recognized mainly by hydrogen bonding (Supplementary Fig. 4b). The Glu478 side chain in OPTN hydrogen bonds with the Arg74 side chain, near the linkage of the linear ubiquitin chain, in the distal ubiquitin (Supplementary Figs 4c and 5a). Glu478 also hydrogen bonds with the side chains of Arg479 and Arg482 in the other OPTN protomer, which in turn respectively hydrogen bond with Gln2 and Glu16 in the proximal ubiquitin (Supplementary Figs 4c and 5a). The Asp474 side chain in OPTN hydrogen bonds with the Arg72 side chain and the main chain nitrogen atom of Leu73 in the distal ubiquitin (Supplementary Fig. 4a). These close recognitions of the linear ubiquitin linkage may explain why the E478G mutation specifically abolished linear ubiquitin-binding. Interestingly, the OPTN residues involved in linear ubiquitin recognition, such as D474 and E478, correlate with those crucial for the suppression of LUBAC- and TNF-α-induced NF-κB activation (Fig. 1b). This suggested that specific recognition of linear ubiquitin by these OPTN-UBAN domain residues is indispensable for suppression of NF-κB activation.

### OPTN suppresses NF-κB activation

To examine the function of OPTN in cells, we constructed *OPTN*-KO cells with the CRISPR/Cas9 system, by targeting exon 5 of the *OPTN* gene. We obtained two lines of *OPTN*-KO cells for both HeLa and BJAB cells, designated as *OPTN*^−/−^-H1 and -H2 for HeLa cells and *OPTN*^−/−^-B1 and -B2 for BJAB cells (Supplementary Fig. 6a). Genome editing of the *OPTN* gene was confirmed by deletion of the restriction enzyme site, nucleotide sequencing, reduced *OPTN* messenger RNA levels and immunoblotting (Supplementary Fig. 6b–e). The *OPTN* deletion did not affect the expression of TNFR and other NF-κB-activating factors such as LUBAC, NEMO and RIP1 (Supplementary Fig. 6e).

**Figure 3:**
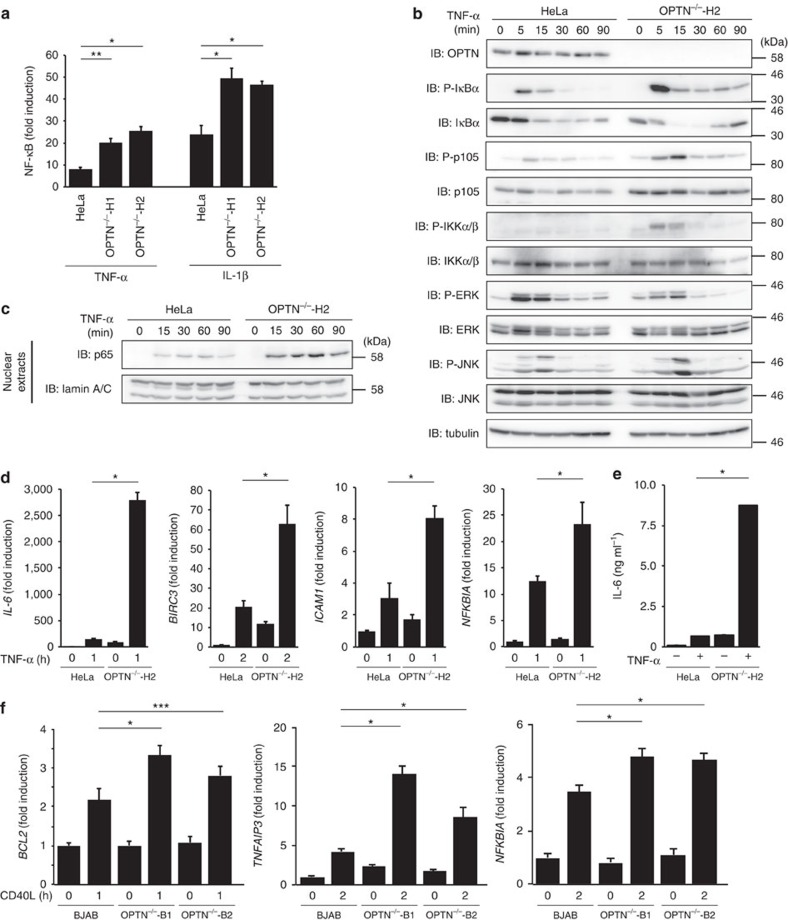
Enhanced NF-κB activation in *OPTN*-KO cells. (**a**) TNF-α- and IL-1β-induced NF-κB luciferase activities in *OPTN*-KO HeLa cells and their parental cells. (**b**) Enhanced NF-κB activation in TNF-α-treated *OPTN*-KO HeLa cells. Cells were treated with TNF-α for the indicated times and immunoblotting was performed using the indicated antibodies. (**c**) Accelerated TNF-α-induced nuclear translocation of p65 in *OPTN*-KO HeLa cells. (**d**) Quantitative PCR (qPCR) analyses of NF-κB targets in TNF-α-treated parental and *OPTN*-KO HeLa cells. Relative expression of target mRNAs was analysed using GAPDH as an internal standard. (**e**) Quantification of secreted IL-6 from TNF-α-treated parental and *OPTN*-KO HeLa cells. (**f**) qPCR analyses of NF-κB targets in CD40L-treated parental and *OPTN*-KO BJAB cells. (**a**,**d**,**e**,**f**) Mean±s.e.m. (*n*=3). Statistical significance was assessed using one-way analysis of variance. *P<0.001, **P<0.01 and ***P<0.05.

Deletion of *OPTN* in HeLa cells enhanced NF-κB reporter activity on TNF-α and interleukin (IL)-1β stimulations (Fig. 3a). Phosphorylation of NF-κB factors such as IκBα, p105 and IKKα/β was also enhanced in *OPTN*-KO HeLa cells stimulated with TNF-α, although mitogen-activated protein kinases were not affected (Fig. 3b). We also performed RNA interference-mediated-*OPTN* knockdown (KD) in HeLa cells and confirmed the increased NF-κB activation on TNF-α stimulation^14^, as shown by IκBα, p105, IKKα/β and p65 phosphorylation (Supplementary Fig. 7a). However, NF-κB activation was more enhanced in *OPTN*-KO HeLa cells, as compared with *OPTN*-KD cells (compare Fig. 3b and Supplementary Fig. 7a). Intranuclear translocation of p65 (Fig. 3c), and induction of NF-κB target genes were also elevated in *OPTN*-KO cells (Fig. 3d,e). Similarly, increases in NF-κB activation and target gene expression were confirmed in IL-1β-treated *OPTN-*KD and *OPTN*-KO HeLa cells (Supplementary Figs 7b and 8). The knock-in of OPTN-WT, but not the E478G mutant, to *OPTN*-KO cells recovered the inhibitory effect on TNF-α-mediated NF-κB activation (Supplementary Fig. 9). Furthermore, NF-κB target gene expression was also enhanced in CD40L-stimulated *OPTN*-KO BJAB cells (Fig. 3f). These results indicated that OPTN physiologically functions as a negative regulator of the canonical NF-κB activation pathway. Thus, the loss of OPTN or the ALS-associated E478G mutation enhanced NF-κB activation.

### OPTN regulates TNFR signalling complex I

**Figure 4:**
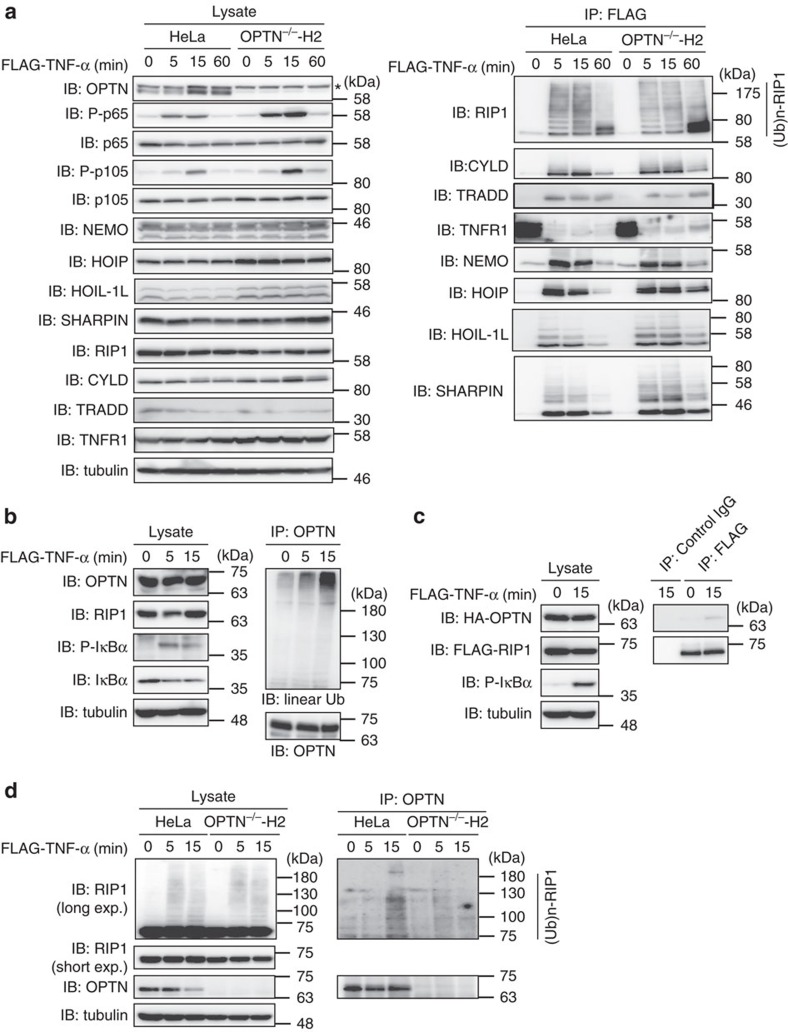
OPTN regulates TNFR signalling complex I. (**a**) Parental and *OPTN-*KO HeLa cells were stimulated with FLAG-TNF-α for the indicated times and then TNFR complex I was immunoprecipitated using an anti-FLAG antibody, followed by immunoblotting using the indicated antibodies. (**b**) Association of endogenous OPTN with free and/or conjugated linear ubiquitin chain on TNF-α stimulation. HEK293T cells were treated with FLAG-TNF-α and anti-OPTN immunoprecipitates were blotted with an anti-linear ubiquitin antibody. (**c**,**d**) Association of OPTN with RIP1 on TNF-α stimulation, examined at overexpressed (**c**) and endogenous (**d**) levels.

On TNF-α stimulation, multiple proteins, such as RIP1, TNFR-associated protein with a death domain (TRADD) and LUBAC, are recruited to form the TNFR signalling complex I, which is crucial for canonical IKK-mediated NF-κB activation^34^. To examine the effect of OPTN on the formation of TNFR signalling complex I, we stimulated *OPTN*-KO HeLa cells and their parental HeLa cells with FLAG-TNF-α (Fig. 4a). NF-κB activation was elevated in *OPTN*-KO cells stimulated with FLAG-TNF-α, as shown by the enhanced phosphorylation of NF-κB factors, such as p65 and p105 (Fig. 4a). Immunoprecipitation by FLAG–TNF-α showed transient association of TNFR with RIP1, CYLD, TRADD, NEMO and LUBAC. Compared with parental cells, association and polyubiquitination of RIP1, HOIL-1L and SHARPIN were enhanced in *OPTN*-KO HeLa cells, indicating that OPTN regulates the association and retention of various molecules to the TNFR complex. In contrast, the overexpression of OPTN suppressed the associations of ubiquitinated RIP1, NEMO and HOIP with TNFR (Supplementary Fig. 10). Collectively, these results indicated that OPTN is a critical regulator of TNFR complex I.

We next searched for proteins that transiently bind to OPTN during TNF-α-induced NF-κB activation. Interestingly, immunoprecipitates of endogenous OPTN from HEK293T cells were highly reactive to the anti-linear ubiquitin antibody on TNF-α stimulation (Fig. 4b). This strongly suggested that OPTN binds free and/or conjugated linear ubiquitin chains on TNF-α stimulation. Ubiquitinated RIP1 bound OPTN^14^ and we confirmed that TNF-α-stimulation indeed enhanced the association of OPTN and RIP1 at both overexpressed and endogenous levels (Fig. 4c,d).

As OPTN shares high homology with NEMO, we investigated whether OPTN could also be linearly ubiquitinated similar to NEMO, and thus have a dominant negative effect. In overexpression experiments with HEK293T cells, OPTN bound to LUBAC components (HOIP and HOIL-1L) and underwent linear ubiquitination (Supplementary Fig. 11a,b). However, we could not detect endogenous linear ubiquitination of OPTN on TNF-α stimulation (Supplementary Fig. 11c). Moreover, conjugation of di- and tetraubiquitins at the carboxy terminus of OPTN, to mimic linearly ubiquitinated OPTN, did not induce NF-κB activation, in contrast to NEMO (Supplementary Fig. 11d)^31^. Overall, our results suggested that although OPTN binds to LUBAC and can be linearly ubiquitinated at overexpressed levels, it is neither a physiological substrate of LUBAC at endogenous levels nor a potential activator of the NF-κB pathway. More importantly, OPTN associates with linearly ubiquitinated proteins, such as RIP1 and NEMO, through its UBAN domain on TNF-α-stimulation, which is important to regulate TNFR complex I.

### OPTN regulates apoptosis

**Figure 5:**
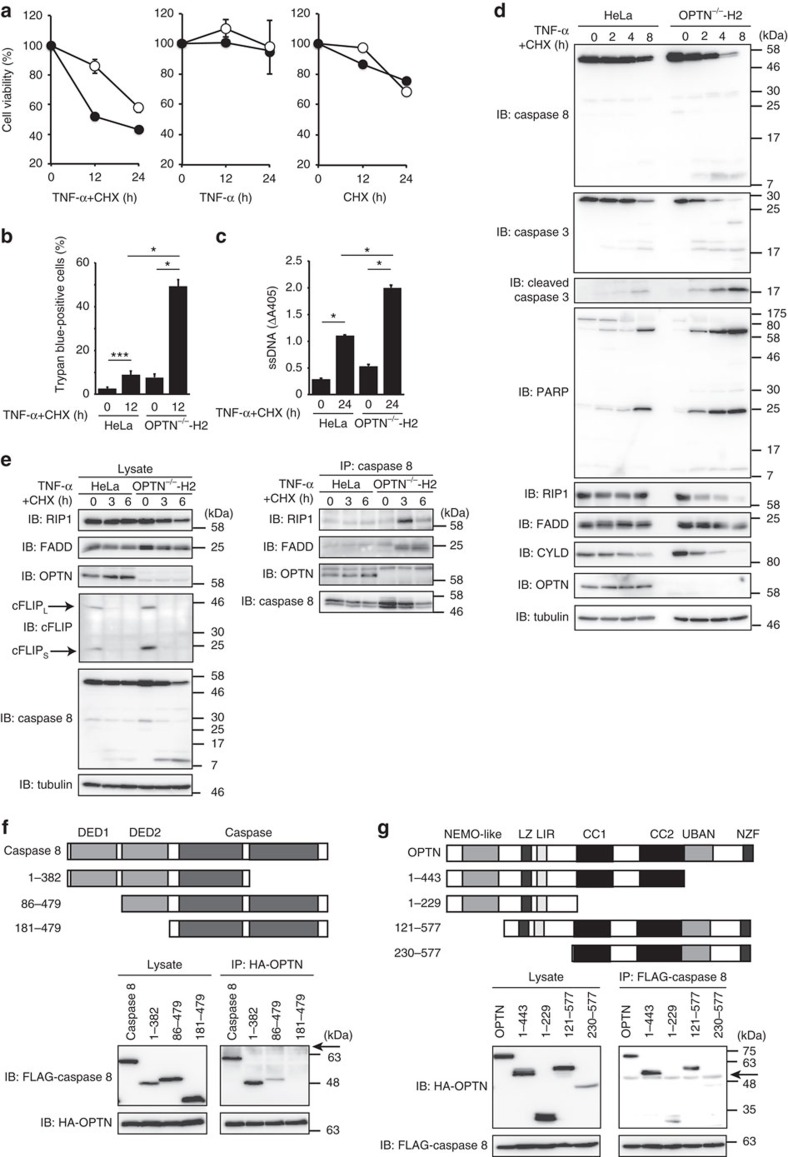
OPTN suppresses TNF-α-induced apoptosis. (**a**) Accelerated cell death in TNF-α- and CHX-treated *OPTN*-KO HeLa cells. Cell survival assays were performed in the presence of TNF-α and/or CHX. Relative cell viabilities of WT (open circles) and *OPTN*-KO (filled circles) HeLa cells in the presence of TNF-α and/or CHX are indicated. Mean±s.e.m. (*n*=3). (**b**) Trypan blue exclusion assay. (**c**) ELISA for cytoplasmic histone-associated DNA fragments. (**b**,**c**) Mean±s.e.m. (*n*=3). Statistical significance was assessed using one-way analysis of variance. *P<0.001, **P<0.01 and ***P<0.05. (**d**) Enhanced cleavage of caspase 8, caspase 3 and PARP in TNF-α- and CHX-treated *OPTN*-KO HeLa cells. Immunoblotting of cell lysates, treated with TNF-α and CHX, was performed using the indicated antibodies. (**e**) Enhanced TNFR complex II formation in TNF-α- and CHX-treated *OPTN*-KO cells. Cells were treated with TNF-α and CHX for the indicated times, and cell lysates and anti-caspase 8 immunoprecipitates were immunoblotted by the indicated antibodies. (**f**,**g**) Identification of interacting regions between caspase 8 and OPTN. Co-immunoprecipitation experiments were performed using various mutants of caspase 8 (**d**) and OPTN (**e**). Arrows, nonspecific signal.

When TNFR signalling complex I fails to activate NF-κB, this leads to the formation of a second complex, named complex II. Complex II induces apoptosis and comprises RIP1, Fas-associated death domain protein (FADD) and pro-caspase 8 (refs 35, 36). As OPTN affected the complex I components on TNF-α stimulation, we next investigated the effect of OPTN on the subsequent TNF-α-induced complex II formation. Combined TNF-α and cycloheximide (CHX) treatments, but not the single treatment with either reagent, induced accelerated cell death in *OPTN*-KO HeLa cells (Fig. 5a–c). In contrast, cell death induced by doxorubicin treatment, which activates the intrinsic mitochondrial apoptosis pathway, was similar in either the presence or absence of OPTN (Supplementary Fig. 12a). Enhanced cleavages of caspase 8, caspase 3, PARP, RIP1 and CYLD were detected in *OPTN*-KO HeLa cells (Fig. 5d). Similarly, enhanced cleavages of caspases 8 and 3 were detected in *OPTN*-KD cells (Supplementary Fig. 12b). Such activation of caspases was inhibited by zVAD-FMK, a pan-caspase inhibitor (Supplementary Fig. 12c). Interestingly, knock-in of OPTN-WT to *OPTN*-KO HeLa cells restored TNF-α-induced caspase activation to levels similar to that of parental HeLa cells, but that of the E478G mutant showed only a partial effect (Supplementary Fig. 12d). These results indicated that OPTN suppresses TNF-α-induced apoptosis.

Although rapid cellular FLICE-like inhibitory protein (cFLIP) turnover was detected at similar levels in both parental and *OPTN*-KO HeLa cells after TNF-α+CHX treatment, the immunoprecipitated caspase 8 revealed the drastically elevated association of RIP1 and FADD to caspase 8 in *OPTN*-KO cells, suggesting that the *OPTN* deletion enhanced complex II formation independently of cFLIP (Fig. 5e). Furthermore, we found that endogenous pro-caspase 8 constitutively associates with OPTN in parental HeLa cells. To identify the interaction domains between caspase 8 and OPTN, we performed co-immunoprecipitation analyses. When overexpressed in HEK293T cells, the full-length active-site mutant of pro-caspase 8 co-immunoprecipitated with full-length OPTN. However, the deletion of the N-terminal death effector domains (DEDs) in caspase 8 abrogated its binding to OPTN (Fig. 5f). In addition, the N-terminal portion of OPTN (residues 121–230), including the leucine zipper and LIR domains, was crucial for caspase 8-binding (Fig. 5g). These results suggested that OPTN physiologically binds to caspase 8 and regulates TNF-α-induced apoptosis, and that the loss of OPTN accelerates complex II formation.

### Linear ubiquitin is involved in OPTN-associated ALS

**Figure 6:**
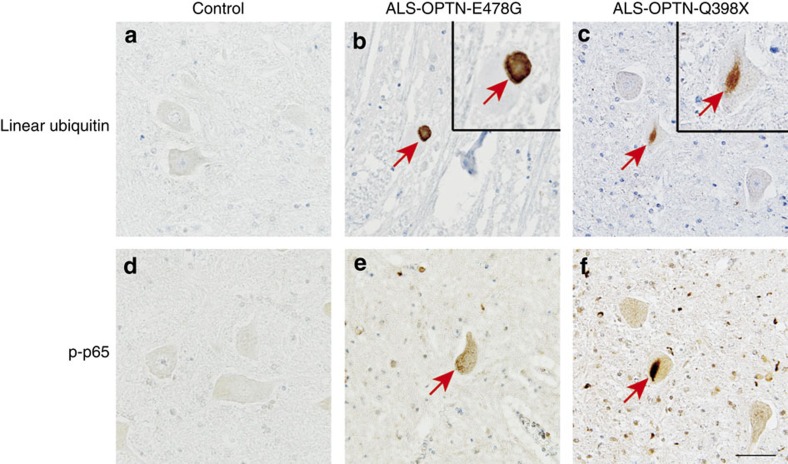
Intracytoplasmic inclusions of OPTN-associated ALS are immunoreactive to linear ubiquitin. Immunohistochemical staining of specimens from a leukaemia patient (control) and ALS patients with the heterozygous OPTN-E478G mutation and the homozygous OPTN-Q398X mutation was performed using anti-linear ubiquitin (**a**–**c**) and anti-P-p65 (**d**–**f**). Arrows indicate immunoreactive inclusions (**b**,**c**,**e**,**f**) in the spinal anterior horn cells. Scale bar, 50 μm.

**Figure 7:**
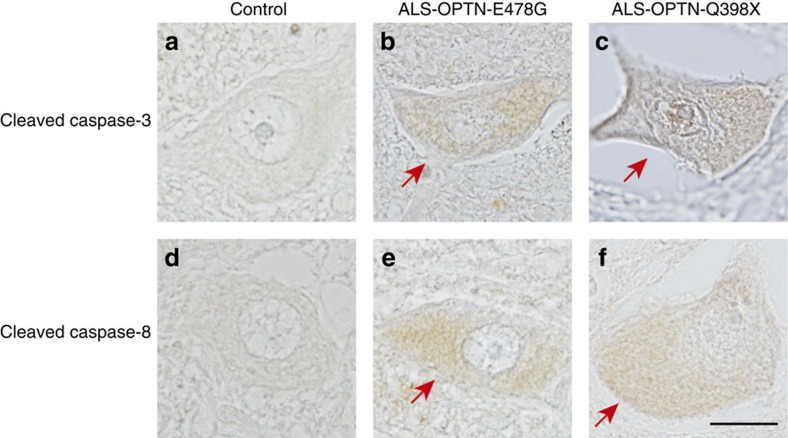
Cleaved caspase 3 and cleaved caspase 8 immunoreactivities are increased in the cytoplasms of spinal anterior horn cells from OPTN-associated ALS cases. Immunohistochemical staining of specimens from a control subject and ALS patients with the heterozygous OPTN-E478G mutation and with the homozygous OPTN-Q398X mutation were performed using anti-cleaved caspase 3 (**a**–**c**) and anti-cleaved caspase 8 (**d**–**f**). Faintly increased diffuse cytoplasmic immunoreactivities of the spinal anterior horn cells from the OPTN-associated ALS patients are indicated by arrows (**b**,**c**,**e**,**f**). Scale bar, 20 μm.

Finally, we investigated whether linear ubiquitin chains were involved in the pathology of OPTN-associated ALS. In ALS patients, ubiquitin- and TDP-43-positive inclusion bodies are reportedly detected in motor neurons of the primary motor cortex, brainstem and spinal cord^24^,^37^,^38^. We examined specimens from an OPTN-associated ALS patient with the heterozygous E478G missense mutation and another patient with the homozygous Q398X nonsense mutation of *OPTN*^24^. Immunohistochemical analyses of the lumbar anterior horn cells revealed intracytoplasmic inclusions staining positive for linear ubiquitin in comparison with control specimens (Fig. 6a–c). To verify whether NF-κB activation was enhanced in OPTN-associated ALS, we stained for phosphorylated p65 (P-p65) and unexpectedly found that P-p65 was also localized in the linear ubiquitin-positive intracytoplasmic inclusions (Fig. 6d–f). We detected partial co-localization of linear ubiquitin and P-p65 with phosphorylated TDP-43 (P-TDP-43) by double immunofluorescent staining analyses (Supplementary Fig. 13). Our preliminary semi-quantitative analyses indicated that the signals from 40% of linear ubiquitin and P-TDP-43 (two cells/5P-TDP-43-positive cells), and 50% of P-p65 and P-TDP-43 (four cells/8P-TDP-43-positive cells) were merged. Furthermore, we performed immunostaining with anti-cleaved caspase 3 and anti-cleaved caspase 8 antibodies (Fig. 7), and detected diffuse staining of these activated caspases in the cytoplasms of the spinal anterior horn cells in the *OPTN*-associated ALS patient samples. Neither activated caspase 3- nor caspase 8-positive cells were found in the control subjects. On the other hand, the percentages of cleaved caspase 3-positive cells among the residual anterior horn cells in ALS patients with the E478G mutation and with the Q398X mutation of *OPTN* were 14.5% (9/62 cells) and 15.9% (7/44 cells), respectively, and those for cleaved caspase 8 were 14.3% (7/49 cells) and 16.7% (7/42 cells), respectively. We could not detect the co-localization of cleaved caspase 3 or cleaved caspase 8 and P-TDP-43, because of the diffuse caspase 3 and caspase 8 staining patterns. Taken together, OPTN deficiency causes the initial enhancement of NF-κB activation but is followed by the impaired intranuclear translocation of NF-κB, eventually leading to an apoptotic milieu induced by increased complex II formation.

## Discussion

In this study, we performed a comprehensive analysis using known OPTN mutants associated with ALS and POAG. Most of the ALS-associated OPTN mutants, but not the POAG-associated mutants, lost the inhibitory effects on TNF-α- and LUBAC-mediated canonical NF-κB activation (Fig. 1). This suggested that impaired NF-κB regulation due to *OPTN* mutations is correlated with ALS pathogenesis. In OPTN-associated POAG, OPTN mutants such as E50K and M98K induce defects in vesicle trafficking and autophagy, and increase retinal ganglion cell death by apoptosis^29^. In contrast, in OPTN-associated ALS, such as the E478G mutation of *OPTN*, perturbations in NF-κB signalling, IRF3 activation, autophagy, mitophagy and intracellular trafficking have been reported^12^,^29^. A recent study using *OPTN*-KO mice indicated that the deficiency of *OPTN* affects IFN signalling and *Salmonella*-induced autophagy, but not NF-κB signalling^39^. However, unlike humans, *OPTN*-KO mice did not exhibit any symptoms resembling glaucoma and ALS. Therefore, it should be noted that differences in species and/or OPTN-related signal transduction pathways might induce neurodegenerative disorders specifically in humans. Our results indicated that linear ubiquitin binding by OPTN-UBAN regulates IKK-mediated NF-κB regulation, but not TBK1/IKKɛ-mediated pathways (Fig. 3 and Supplementary Figs 1,2 and 6–9).

In MBP pull-down assays, linear ubiquitin binding was greatly diminished by the E478G mutation, as compared with that of OPTN-WT (Fig. 2a), and the *K*_D_ value of full-length OPTN-WT to linear ubiquitin was determined to be 1.0 μM (Fig. 2b), which was close to that of NEMO-UBAN binding to linear ubiquitin^9^,^32^. Crystal structure analyses revealed that the OPTN-UBAN domain selectively binds to the linear ubiquitin chain in a similar manner to the NEMO-UBAN domain^9^, and that the ALS-associated E478 residue is essential for specific linear ubiquitin recognition, but not K63-linked ubiquitin, as no important interaction seems to occur between E478 and K63-linked ubiquitin, as deduced from the structure of NEMO-UBAN K63 diubiquitin (Fig. 2c–e and Supplementary Fig. 5). Our results strongly suggested that ALS-associated mutations of OPTN-UBAN cause defects in linear ubiquitin-binding ability and, consequently, critically affect the pathogenesis of ALS by causing dysregulation of IKK-mediated NF-κB activation.

In addition to linear ubiquitin-binding, we previously showed that NEMO itself is linearly ubiquitinated at residues K285 and K309 by LUBAC on NF-κB activation^6^. As the structures of OPTN-UBAN and NEMO-UBAN are quite similar (Fig. 2d), we tested whether OPTN could also be linearly ubiquitinated by binding to LUBAC on overexpression (Supplementary Fig. 11). OPTN was linearly ubiquitinated on overexpression, but not at endogenous levels, suggesting that unlike NEMO, OPTN is not a preferential substrate of LUBAC. This may be explained by differences in the LUBAC affinity between NEMO and OPTN. LUBAC recognizes the CC2 region of NEMO, including amino acid residues from Gln259 to Asp275, via the Npl4-type zinc finger-1 domain of HOIP^10^. Differences in the coiled-coil conformation and/or local amino acid residues between NEMO and OPTN may affect the LUBAC affinity. Thus, we concluded that OPTN functions as a linear ubiquitin-binding protein through its UBAN domain, and that linearly ubiquitinated proteins, such as RIP1 and NEMO, are important candidates of OPTN-binding. Indeed, in CRISPR/Cas9-mediated *OPTN*-KO HeLa cells, immunoprecipitation of TNF-α revealed prolonged association and polyubiquitination of TNFR complex I components, such as RIP1, NEMO and LUBAC (Fig. 4a), whereas the overexpression of OPTN showed the opposite reaction (Supplementary Fig. 10). Thus, these results suggested that OPTN affects the ubiquitination and association of TNFR complex I components such as RIP1 and NEMO by competing through its linear ubiquitin-binding UBAN domain.

In the absence of OPTN, complex II formation was accelerated during the extrinsic apoptosis pathway induced by TNF-α and CHX treatments (Fig. 5a–e). OPTN also physiologically associated with caspase 8 at the DED domains (Fig. 5e,f), which are important regions for the recruitment of other DED domain-containing proteins, such as FADD^40^. As the deubiquitination of RIP1 by CYLD, a major deubiquitinase in the NF-κB pathway that functions by hydrolysing K63 and linear ubiquitin chains^41^, is a key step to induce complex II formation^42^ and OPTN associates with CYLD^43^, defects in CYLD deubiquitination activity, due to the absence of OPTN, may result in enhanced complex II formation.

ALS is a fatal neurodegenerative disorder affecting large motor neurons of the brain and spinal cord. Protein/RNA aggregate formation, failures in clearance by proteasomes and autophagy, and neuroinflammation by the NF-κB and IFN signalling pathways play crucial roles in the molecular pathogenesis of ALS^25^. OPTN-associated ALS is rare (<1%) in familial and sporadic ALS^25^, but the patients have intracytoplasmic inclusions in the anterior horn cells and immunoreactivity for ubiquitin and TDP-43 (refs 24, 38). We found that these intracytoplasmic inclusions, in the spinal cord of ALS patients with the heterozygous OPTN-E478G or homozygous Q398X mutation, stained positive for linear ubiquitin and activated NF-κB (P-p65) in partial co-localization with P-TDP-43 (Fig. 6 and Supplementary Fig. 13). A recent report demonstrated that the co-localization of TDP-43 and p65 disturbs the nuclear translocation of p65, thus also supporting the involvement of NF-κB dysregulation in ALS^44^. Moreover, cleaved caspase 3 was also detected in the OPTN-associated ALS patients, suggesting enhanced apoptosis in the absence of OPTN. We previously reported that the *Optn*-KD in Neuro2a cells increases apoptosis^45^. Although restoration of the WT and POAG-associated E50K OPTN mutant suppressed cell death, ALS-associated OPTN mutants such as Q398X and E478G could not, suggesting that apoptosis is also pivotal in ALS pathogenesis.

OPTN has recently attracted much attention as a receptor/regulator of autophagy. First, OPTN reportedly regulates autophagic clearance of ubiquitin-coated cytosolic *Salmonella* in infected HeLa cells, through phosphorylation of OPTN-LIR by TBK1 (ref. 17). OPTN also recognizes protein aggregates during autophagy^46^. As for mitophagy, OPTN and NDP52 have been linked to PINK1 (PTEN-induced putative kinase 1)- and parkin-mediated mitophagy, and OPTN-UBAN is required for this process^18^,^19^,^20^. Phosphorylations of Ser65 in both ubiquitin and parkin are critical triggers for mitophagy and are involved in the pathophysiology of Parkinson's disease^47^; however, the effect of ubiquitin phosphorylation on the interaction between ubiquitin and OPTN remains controversial. One report found that OPTN exhibits higher affinity to the phosphomimetic S65D ubiquitin than to WT ubiquitin^19^, whereas others reported that OPTN shows lower affinity to phosphorylated K63- and M1-linked ubiquitins, as compared with unphosphorylated ubiquitin^20^,^48^. Our structural analysis revealed that Ser65, in both the distal and proximal ubiquitins in the linear ubiquitin chain, does not directly interact with OPTN-UBAN (Fig. 2c and Supplementary Fig. 14). Recently, TBK1 was found to phosphorylate Ser473 and Ser513 of OPTN, in addition to the previously identified Ser177, and the phosphorylation of Ser473 plays a major role to promote ubiquitin binding^20^. Our structural analysis suggested that Ser473 of OPTN hydrogen bonds with Arg74 of the distal ubiquitin and thus the phosphorylation of this residue might affect the ubiquitin binding of OPTN (Fig. 2c and Supplementary Fig. 14).

Defects in autophagy and mitophagy result in the accumulation of misfolded proteins and damaged organelles, in a hypothetical model of ALS pathogenesis^49^. Interestingly, our results showed that intracytoplasmic inclusions in OPTN-associated ALS patients stained positive for linear ubiquitin chain (Fig. 6), suggesting that linear ubiquitination is involved in the molecular pathogenesis of ALS. We confirmed the role of OPTN as a regulator of NF-κB activation and apoptosis, especially in the context of OPTN-associated ALS. Taken together, we speculate that OPTN, with its linear ubiquitin-binding ability, stands at the cross-roads of NF-κB activation, apoptosis and possibly autophagy, resulting in the complex and multifactorial pathogenesis of ALS.

## Methods

### Plasmids

The open reading frames of human OPTN, caspase 8 and the other complementary DNAs^6^,^8^ were amplified by reverse transcription–PCR. Mutants of these cDNAs were prepared by the QuikChange method and the whole nucleotide sequences were verified. The cDNAs were ligated to the appropriate epitope sequences and cloned into the pcDNA3.1 (Invitrogen) and pMAL-c2x (New England Biolabs) vectors.

### Antibodies

The following antibodies were used for immunoblotting and full-size images for immunoblottings are presented in Supplementary Fig. 15. Catalogue number and dilutions are indicated in the parentheses. P-IκBα (9246; 1:1,000), IκBα (4812; 1:1,000), P-p105 (4806; 1:1,000), p105 (13586; 1:1,000), P-p65 (3033; 1:1,000), p65 (8242; 1:1,000), P-IKKα/β (2697; 1:1,000), P-JNK (4668; 1:1,000), JNK (5668; 1:1,000), P-ERK (4370; 1:2,000), ERK (4695; 1:2,000), caspase 8 (4790; 1:1,000), cleaved caspase 8 (Asp391) (9496; 1:1,000), caspase 3 (9662; 1:1,000), cleaved caspase 3 (9661; 1:1,000), PARP (9542; 1:1,000), CYLD (8462; 1:1,000), TBK1 (3504; 1:1,000) and Lamin A/C (4777; 1:2,000) were obtained from Cell Signaling. OPTN (clone C-2, sc-166576; 1:1,000), ubiquitin (clone P4D1, sc-8017; 1:1,000), TNFR1 (clone H271, sc-7895; 1:1,000), IKKα/β (clone H-470, sc-7607; 1:1,000), TRADD (sc-46653; 1:1,000) and NEMO (clone FL-419, sc-8330; 1:1,000) were purchased from Santa Cruz Biotechnology. RIP1 (610458; 1:1,000) and FADD (610399; 1:1,000) were from BD Transduction Laboratories. OPTN (A301-829A, Bethyl and Proteintech; 1:1,000), linear ubiquitin (clone LUB9, MABS451, Millipore; 1:1,000), tubulin (CLT9002, Cedarlane; 1:3,000), NEMO (clone EA2-6, K0159-3, MBL; 1:1,000), HOIP (SAB2102031, Sigma-Aldrich; 1:1,000), HOIL-1L (NBP1-88301, Novus Biologicals; 1:1,000) and SHARPIN (14626-1-AP, Proteintech; 1:1,000) were also used.

### Cell culture and luciferase assay

HEK293T and HeLa cells (ATCC) were cultured in DMEM containing 10% fetal bovine serum, 100 IU ml^−1^ penicillin G and 100 μg ml^−1^ streptomycin, and BJAB cells (ATCC) were maintained in RPMI containing 10% fetal bovine serum and antibiotics, at 37 °C under 5% CO_2_. Transfection experiments were performed using Lipofectamine 2000 or TurboFect (Thermo Fisher). For KD experiments, control siRNA (sc-37007) and *OPTN* siRNA (sc-39054), obtained from Santa Cruz Biotechnology, were transfected by Lipofectamine RNAiMAX (Thermo Fisher).

The pGL4-IFNβ-promoter-Luc or pGL4-NF-κB-Luc plasmid was co-transfected into HEK293T or HeLa cells with the pGL4-*Renilla*-Luc/TK plasmid^50^. At 24 h after transfection, the cells were lysed and the luciferase activity was measured in a GloMax 20/20 luminometer (Promega), using the Dual-Luciferase Reporter Assay System (Promega).

### MBP pull-down assay

MBP-tagged full-length OPTN and its mutants (E478G, Q398X), full-length NEMO and MBP-LacZ were expressed in *Escherichia coli* Rosetta 2 (DE3) (Novagen) and purified using amylose resin (New England Biolabs). To examine the ubiquitin-binding ability, MBP-fusion proteins (0.8 μM) and tetraubiquitin (2 μM) were incubated in reaction buffer (50 mM Tris-HCl pH 7.5, 150 mM NaCl, 1 mM dithiothreitol, 0.1% NP-40 and 250 μg ml^−1^ BSA) at 37 °C for 1 h, followed by the addition of amylose resin^51^. The samples were further incubated at 4 °C for 1 h with gentle rotation and then the beads were washed three times with the buffer without BSA and analysed by SDS–PAGE. The bound ubiquitin was detected by immunoblotting using an anti-ubiquitin antibody and MBP fusion proteins were stained with Coomassie Brilliant Blue.

### SPR analysis

The kinetic analysis was conducted with a Biacore X100 apparatus (GE Healthcare). A CM5 sensor chip and HBS-EP+ running buffer (10 mM Hepes-NaOH pH 7.4, 150 mM NaCl, 0.05% Tween 20 and 3 mM EDTA) were used. The temperature of the flow cells was kept at 25 °C. Linear tetraubiquitin and BSA were respectively immobilized on the measuring cell and the reference cell by amine coupling. The immobilization level of linear tetraubiquitin was around 500 RU. In each cycle, a different concentration of OPTN protein diluted by HBS-EP+ buffer was injected into the flow cells, at 30 μl min^−1^ for 120 s. The dissociation time was 300 s. Finally, the sensor chip was regenerated by injecting 10 mM NaOH, for 30 s at 10 μl min^−1^. Data analysis was conducted with the BIAevaluation software.

### Crystallography

The gene encoding human OPTN CC2 and UBAN domain (residues 416–510) was cloned into the pGEX-6P vector (GE Healthcare). The glutathione *S*-transferase-tagged OPTN CC2-UBAN protein was overexpressed in *E. coli* Rosetta 2 (DE3) and purified with glutathione Sepharose 4B resin, followed by on-column cleavage of glutathione *S*-transferase using Turbo3C (HRV3C) protease (Accelagen). The eluted protein was further purified by Resource Q column chromatography (GE Healthcare), followed by Superdex 75 column chromatography (GE Healthcare). Linear tetra-ubiquitin was expressed in *E. coli* and purified by heat treatment and ion exchange chromatography using a HiTrap SP column (GE Healthcare), followed by Superdex 200 column chromatography (GE Healthcare)^51^. The final concentrations of the OPTN and tetraubiquitin stock solutions were 3.8 and 6.2 mg ml^−1^, respectively. Crystals were obtained within 1 week at 20 °C by the hanging drop vapour diffusion method, by mixing 1 μl protein solution and 1 μl reservoir solution (16% PEG 3350 and 250 mM HCOOK). The crystals were cryoprotected in the reservoir solution supplemented with 25% ethylene glycol and flash cooled in liquid nitrogen. X-ray diffraction experiments were performed at beamline BL41XU at SPring-8 (Hyogo, Japan), at 1 Å wavelength and 100 K.

The data set was processed with XDS^52^. The structure was determined by sequential molecular replacement, using Phaser^53^ and MOLREP^54^. The first molecular replacement search was performed using Phaser^53^, with the linear diubiquitin and a polyalanine model of the NEMO CC2-UBAN dimer taken from the NEMO-linear diubiquitin complex structure (PDB: 2ZVN) as search models. Two copies of diubiquitin and two copies of the CC2-UBAN dimer were successfully placed. The solution was fixed and the remaining two linear diubiquitin molecules were placed using MOLREP^54^. Manual model building and refinement were performed using COOT^55^ and PHENIX^56^, respectively. A representative electron density map is shown in Supplementary Fig. 4c. In the Ramachandran plot, 95.5, 4.4 and 0.1% of the residues were in the favoured, allowed and outlier regions, respectively. Data collection and refinement statistics are summarized in Table 1.

### Construction of *OPTN*-KO cells

The gRNA_cloning vector (41824) and pCAG-hCas9 (51142) were obtained from Addgene. The nucleotide sequence 5′-CCcacgagaacagtctccactg-3′ in exon 5 of the human *OPTN* gene was selected as the target for gRNA. These plasmids and a puromycin-resistant vector (pXS-Puro) were co-transfected into HeLa and BJAB cells. Two days after transfection, cells were selected with puromycin for 2 days and then cell colonies were obtained by limiting dilution. Genome editing of the *OPTN* gene was screened by PCR and *Bss*SI digestion assays, and nucleotide mutations were confirmed by sequencing.

### Quantitative PCR and ELISA

Cell lysis, reverse-transcription and quantitative PCR were performed with SuperPrep Cell Lysis, RT Kit for quantitative PCR and Power SYBR Green PCR Master Mix (Life Technologies), respectively, according to the manufacturer's instructions. Quantitative real-time PCR was performed with a Step-One-Plus PCR system (Applied Biosystems) by the ΔΔCT method, using the following oligonucleotides: IL-6 sense, 5′-AGCCACTCACCTCTTCAGAAC-3′ and IL-6 anti-sense, 5′-GCCTCTTTGCTGCTTTCACAC-3′; BIRC3 sense, 5′-AGATGAAAATGCAGAGTCATCAAT-3′ and BIRC3 anti-sense, 5′-CATGATTGCATCTTCTGAATGG-3′; ICAM1 sense, 5′-GTGGTAGCAGCCGCAGT-3′ and ICAM1 anti-sense, 5′-TTCGGTTTCATGGGGGT-3′; NFKBIA sense, 5′-CGGGCTGAAGAAGGAGCGGC-3′ and NFKBIA anti-sense, 5′-ACGAGTCCCCGTCCTCGGTG-3′; BCL-2a sense, 5′-CAGGAGAATGGATAAGGCAAA-3′ and BCL-2a anti-sense, 5′-CCAGCCAGATTTAGGTTCAAA-3′; TNFAIP3 sense, 5′-CATGCATGCCACTTCTCAGT-3′, and TNFAIP3 anti-sense, 5′-CATGGGTGTGTCTGTGGAG-3′; and GAPDH sense, 5′-AGCAACAGGGTGGTGGAC-3′ and GAPDH anti-sense, 5′-GTGTGGTGGGGGACTGAG-3′. Secreted human IL-6 was measured by using an ELISA kit (eBioscience).

### TNFR1 complex I analysis

TNFR complex formation on TNF-α stimulation was analysed using FLAG-tagged TNF-α^51^. Briefly, HeLa cells were stimulated for the indicated times with 1 μg ml^−1^ FLAG-tagged TNF-α and then lysed in 1 ml lysis buffer (50 mM Tris-HCl pH 7.5, 150 mM NaCl, 1% Triton X-100 and complete protease inhibitor cocktail) for 15 min on ice. Lysates were immunoprecipitated with 15 μl anti-FLAG M2 beads (Sigma) overnight at 4 °C. The beads were recovered by centrifugation, washed eight times with 1 ml of lysis buffer and then analysed by SDS–PAGE and immunoblotting.

### Cell survival assay

The number of viable cells was measured with a CellTiter 96 AQueous Non-Radioactive Cell Proliferation Assay kit (Promega), based on an MTS (3-(4,5-dimethylthiazol-2-yl)-5-(3-carboxymethoxyphenyl)-2-(4-sulfophenyl)-2H-tetrazolium) compound-based colorimetric assay and by the trypan blue exclusion assay. Moreover, cytoplasmic histone-associated DNA fragments (mono- and oligonucleosomes) generated after induced cell death were measured by a cell-death detection ELISA (Roche), according to the manufacturer's instructions. zVAD-FMK (BD Biosciences) was used as a pan-caspase inhibitor.

### Patient information

The patient with the proband of homozygous recessive OPTN-Q398X developed with the onset of dysarthria, muscle weakness and increased deep tendon reflex at 52 years of age^24^,^37^. On the other hand, the patient with the proband of heterozygous dominant OPTN-E478G showed muscle weakness, dysarthria, increased deep tendon reflex and tongue atrophy at 56 years of age^24^,^38^.

### Immunohistochemistry

For neuropathological examinations, formalin-fixed paraffin-embedded 6 μm-thick sections were deparaffinized, autoclaved and incubated overnight with a given primary antibody, such as anti-linear ubiquitin (clone LUB9, MABS451, Millipore; 1:100), anti-phospho-p65 (pSer536) (600-401-265, Rockland; 1:200), anti-cleaved caspase 3 (9661, Cell Signalling; 1:100) and anti-cleaved caspase 8 (9496, Cell Signalling; 1:50). Bound antibodies were detected by a Vectastain Elite ABC kit (Vector Laboratories), with 3,3'-diaminobenzidine tetrahydrochloride as the chromogen^38^. For double immunofluorescence staining, rabbit polyclonal anti-phospho-TDP-43 antibody (pSer409) (TIP-PTD-P03, Cosmo Bio; 1:2,000) and a mouse monoclonal anti-phospho-TDP-43 antibody (pSer 409/410) (TIP-PTD-M01, Cosmo Bio; 1:2,000) were treated with anti-linear ubiquitin and P-p65 antibodies, respectively. The primary antibodies were detected with Alexa Fluor 488- (Donkey anti-mouse, A21202; Donkey anti-rabbit, A21200; Molecular Probes; 1:200) or Alexa Fluor 546-conjugated secondary antibodies (Donkey anti-mouse, A10036; Donkey anti-rabbit, A10040; 1:200) and observed with an FLUOVIEW FV-1000 confocal laser scanning microscope (Olympus)^38^. Procedures involving the use of human materials were performed in accordance with ethical guidelines set by Wakayama Medical University and Declaration of Helsinki. For quantitative study of these activated caspase-positive cells, we obtained four to six distinct sections from each case for each antibody and two examiners, who had no information regarding the clinical condition of the individual providing the spinal specimen, made observations. Only anterior horn cells with a distinct nucleus and nucleolus were counted.

### Statistical analysis

One-way analysis of variance followed by a *post-hoc* Tukey's honest significant difference test was performed, using the KaleidaGraph software. For all tests, a *P*-value of <0.05 was considered statistically significant.

### Data availability

The coordinates and structure factors of OPTN CC2-UBAN region in complex with linear tetraubiquitin have been deposited in the Protein Data Bank under the accession codes 5B83. The authors declare that data supporting the findings of this study are available within the article and its Supplementary Information files or are available from the corresponding authors on request.

## Additional information

**Accession code:** Referenced accessions; Protein Data Bank; 5B83.

**How to cite this article:** Nakazawa, S. *et al*. Linear ubiquitination is involved in the pathogenesis of optineurin-associated amyotrophic lateral sclerosis. *Nat. Commun.* 7:12547 doi: 10.1038/ncomms12547 (2016).

## Supplementary Material

Supplementary Information Supplementary Figures 1-15.

## Figures and Tables

**Table 1:** Data collection and refinement statistics.

	OPTN/tetraubiquitin
*Data collection*	
Space group	*P*2_1_2_1_2_1_
Cell dimensions	
*a*, *b*, *c* (Å)	71.3, 82.0, 244.9
*α*, *β*, *γ* (°)	90.0, 90.0, 90.0
Resolution (Å)	50–2.69 (2.86–2.69)*
*R*_merge_ (%)	14.1 (92.0)
*I*/*σI*	12.6 (2.24)
CC_1/2_ (%)	99.7 (70.4)
Completeness (%)	99.4 (96.3)
Redundancy	7.3 (7.3)
	
*Refinement*	
Resolution (Å)	49.06–2.69
No. reflections	40,445
*R*_work_/*R*_free_ (%)	20.1/25.4
No. atoms	
Protein	6643
Water	59
*B*-factors (Å^2^)	
Protein	63.08
Water	37.27
Root mean square deviations	
Bond lengths (Å)	1.13
Bond angles (°)	0.010

OPTN, optineurin.

^*^Highest-resolution shell is shown in parenthesis. X-ray data were collected from a single crystal.
