# Supplementary material for: Linear ubiquitination is involved in the pathogenesis of optineurin-associated amyotrophic lateral sclerosis
Source: Nat Commun. 2016 Aug 24;7:12547. doi: 10.1038/ncomms12547 (PMC4999505; doi:10.1038/ncomms12547)
Supplement: Supplementary Information — Supplementary Figures 1-15. [file ncomms12547-s1.pdf]

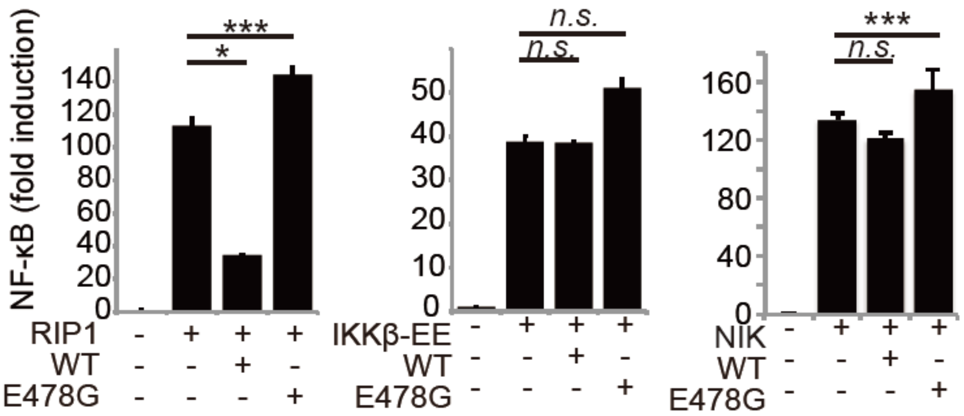

Supplementary Figure 1. Effects of OPTN-WT and E478G mutant on NF-κB activation induced by RIP1, constitutively active mutant of IKKβ, and NIK. The NF-κB luciferase assay was performed in HEK293T cells, and induction folds of NF-κB activity are shown as mean ± s.e.m. (*n* = 3). *: *P*<0.001, ***: *P*<0.05, *n.s.*: not significant.

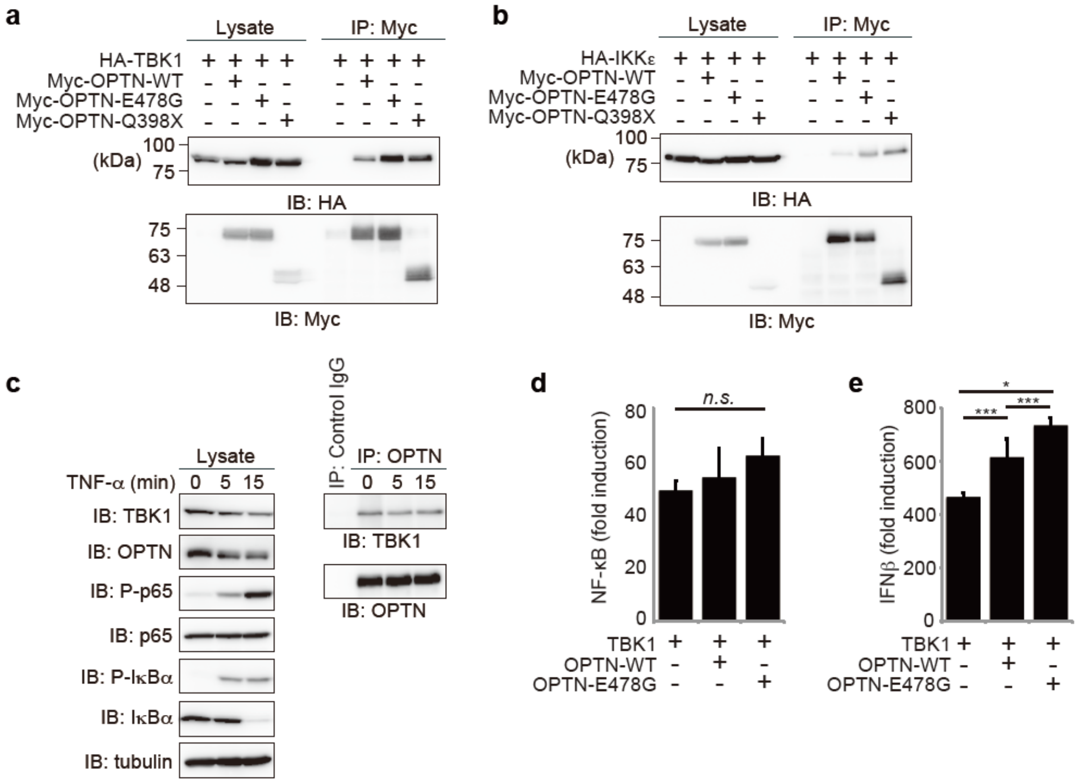

Supplementary Figure 2. Interaction of OPTN-WT and -mutants with TBK1/IKKε, and effects on NF-κB and IFN activation. HEK293T cells transiently expressing HA-TBK1 (**a**) or HA-IKKε (**b**) with Myc-OPTN-WT or -mutants were immunoprecipitated and immunoblotted, as indicated. (**c**) The endogenous interaction of OPTN and TBK1 was not affected by TNF-α stimulation. Cell lysates and immunoprecipitates from HEK293T cells were blotted as indicated. The effects of OPTN-WT and -E478G mutant on TBK1-induced NF-κB (**d**) and IFNβ (**e**) activation were examined by luciferase assays in HEK293T cells, and induction folds are shown as mean ± s.e.m. (*n* = 3). *: *P*<0.001, ***: *P*<0.05, *n.s.*: not significant.

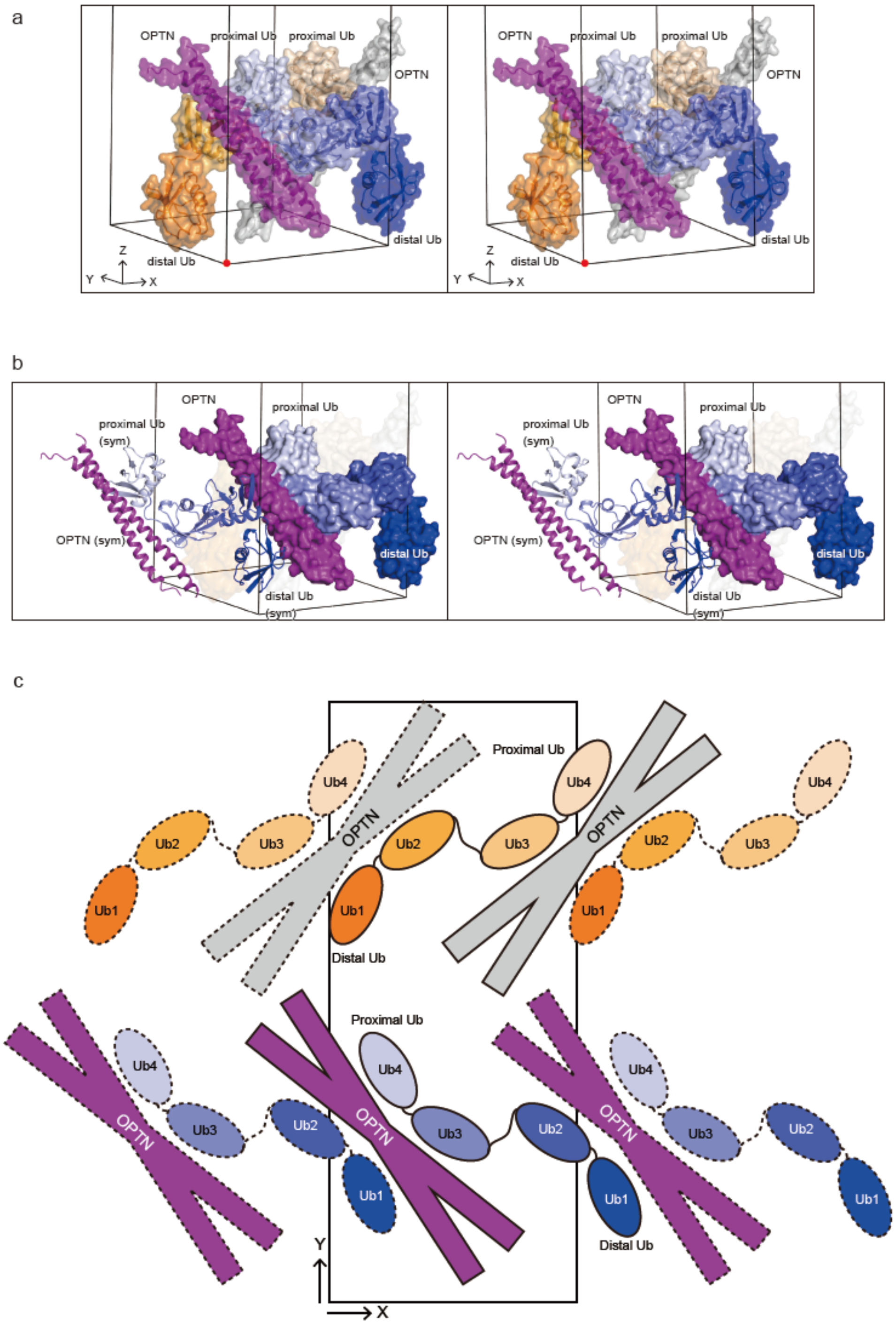

Supplementary Figure 3. Crystal packing of OPTN and tetraubiquitin molecules. (**a**) The asymmetric unit contains two OPTN dimers and two tetraubiquitin molecules (stereo view). The two OPTN dimer molecules are

shown as *purple* and *grey* semi-transparent surfaces with ribbon models, respectively. The two tetraubiquitin molecules are shown as gradient-colored semi-transparent surfaces (*orange* [distal] to *wheat* [proximal] and *blue* [distal] to *light blue* [proximal], respectively) with ribbon models. (**b**) The biological unit of the OPTN-linear ubiquitin complex (stereo view). OPTN binds to the two ubiquitin moieties in the same asymmetric unit on one side and the two ubiquitin moieties from the adjacent unit cell (shown as ribbon models) on the other side. (**c**) Schematic drawings of the crystal packing. Molecules in one asymmetric unit are depicted with *solid lines* and those in adjacent cells are depicted with *dashed lines*.

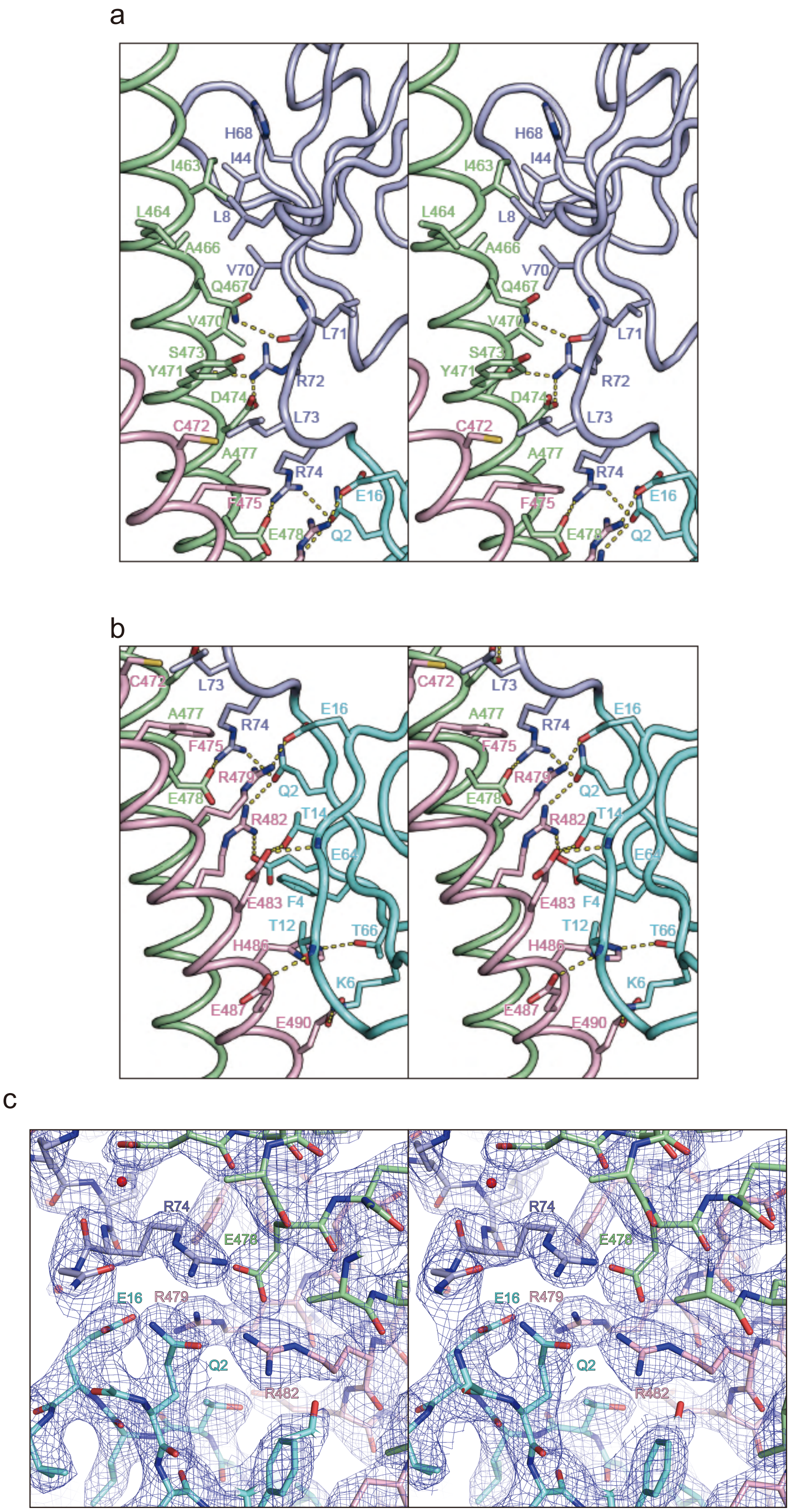
a
H68
I44
I463
L464
L8
A466
V70
Q467
V470
L71
S473
Y471
R72
D474
L73
C472
A477
R74
F475
E16
E478
Q2
b
C472
L73
A477
R74
E16
F475
R479
Q2
E478
R482
T14
E64
E483
F4
T12
T66
H486
K6
E487
E490
c
R74
E478
E16
R479
Q2
R482

Supplementary Figure 4. Stereo view of the interaction between OPTN-UBAN and linear ubiquitin. OPTN and diubiquitin are colored as in Fig. 2c. (**a**) Interactions between OPTN-UBAN and distal ubiquitin. (**b**) Interactions between OPTN-UBAN and proximal ubiquitin. (**c**) Stereoview of the 2Fo-Fc map of the interaction region between OPTN-UBAN and linear ubiquitin contoured at the 1.5σ.

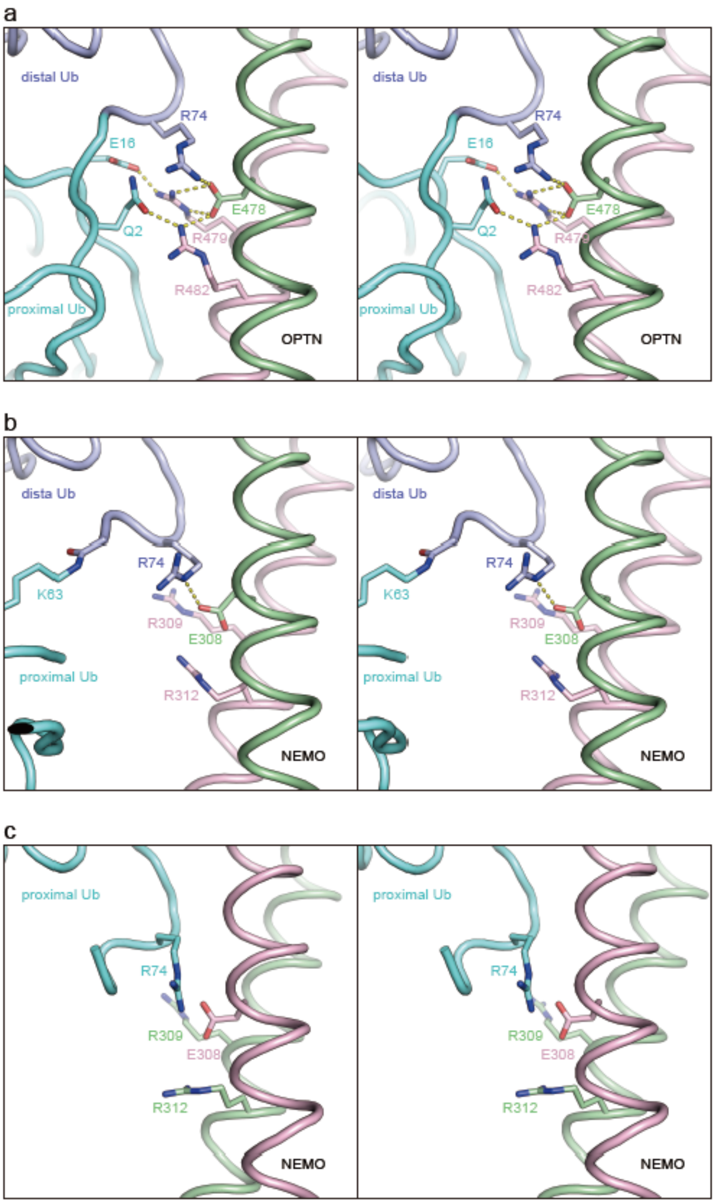

Supplementary Figure 5. Role of E478 of OPTN and the corresponding E308 of NEMO on K63-linked ubiquitin-binding. (**a**) Interactions between OPTN-UBAN and linear ubiquitin. (**b, c**) Interactions between NEMO-UBAN and the distal (**b**) and proximal (**c**) ubiquitins of K63-linked ubiquitin.

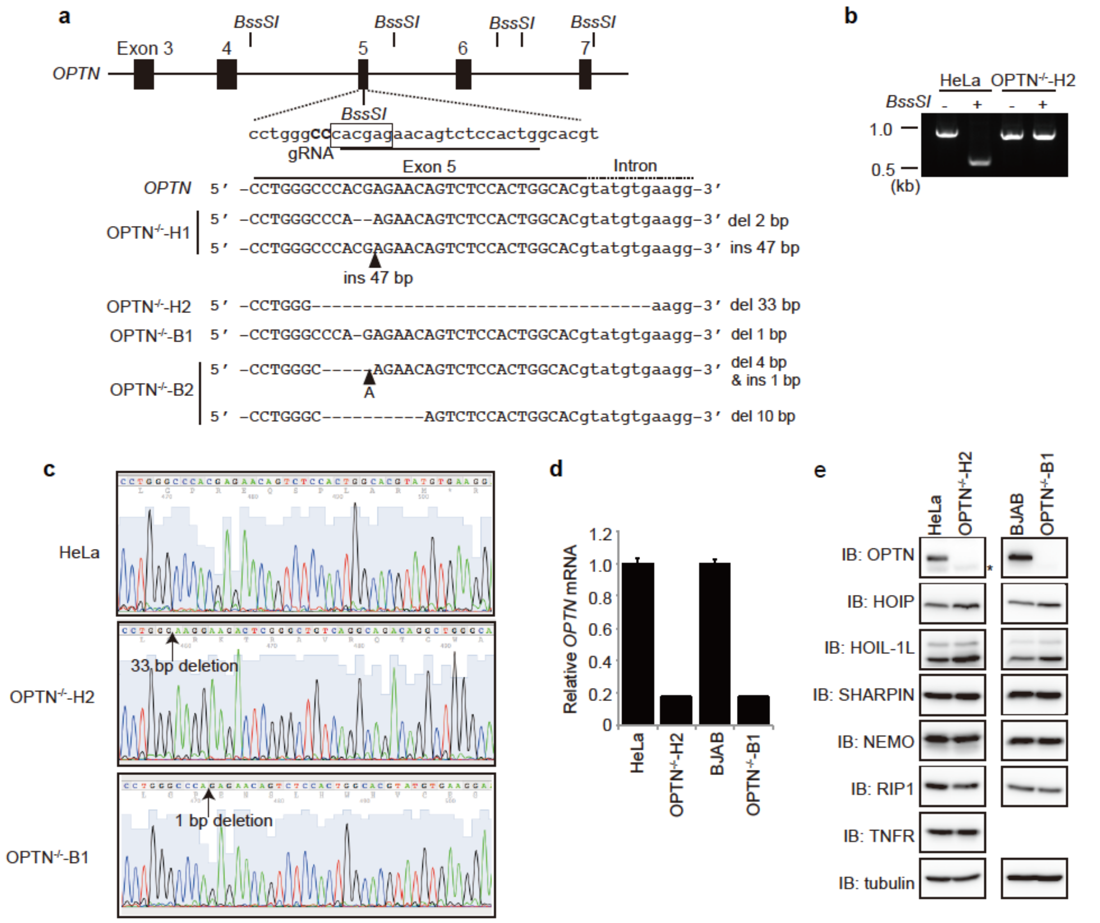

Supplementary Figure 6. Construction of *OPTN*-KO HeLa and BJAB cells by CRISPR/Cas9 technology. (**a**) Scheme for *OPTN*-KO targeting. gRNA was targeted at exon 5 of the human *OPTN* gene. Nucleotide sequences of two cell lines each for *OPTN*-KO HeLa (H1 and H2) and BJAB (B1 and B2) are shown. (**b**) Elimination of the restriction enzyme site in *OPTN*-KO HeLa cells. PCR followed by *Bss*SI digestion was performed to verify *OPTN* gene editing. (**c**) PCR and direct sequence of *OPTN*-KO cells. (**d**) qPCR analyses for mRNA levels of *OPTN* in parental and knockout cells. (**e**) Immunoblotting of *OPTN*-KO and its parental HeLa and BJAB cells.

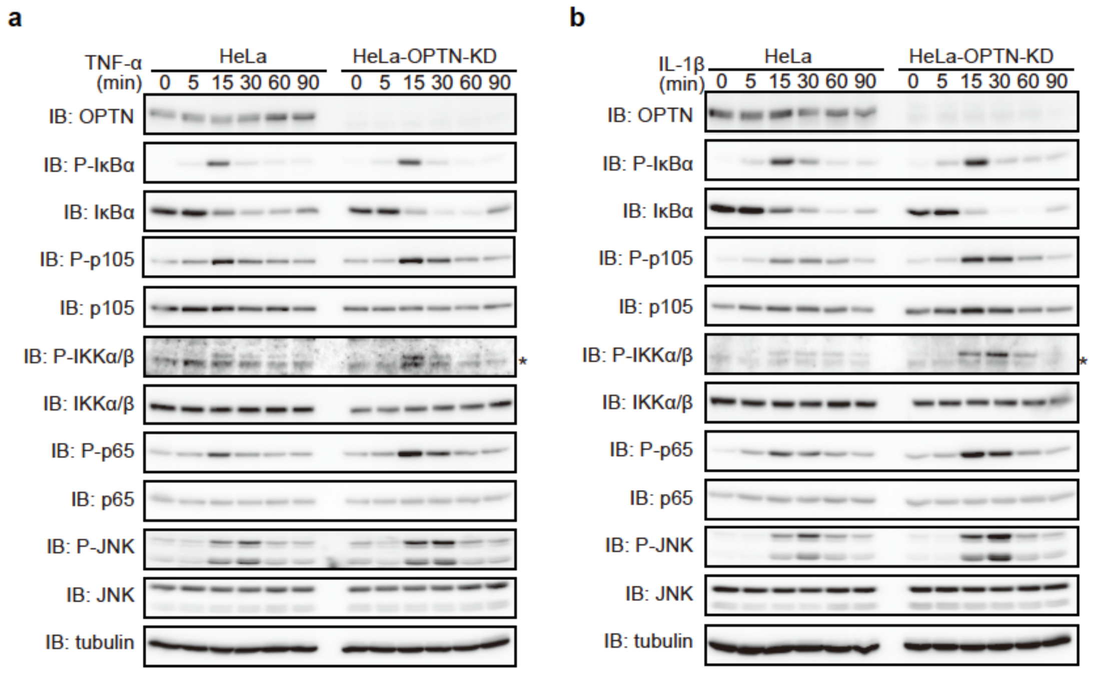

Supplementary Figure 7. Knockdown of *OPTN* causes enhanced NF-κB activation upon proinflammatory cytokine stimulation. HeLa cells were transfected with control or siRNA for *OPTN*, and NF-κB activation after TNF-α- (**a**) or IL-1β- (**b**) treatments was examined by immunoblotting with the indicated antibodies. *; non-specific signal.

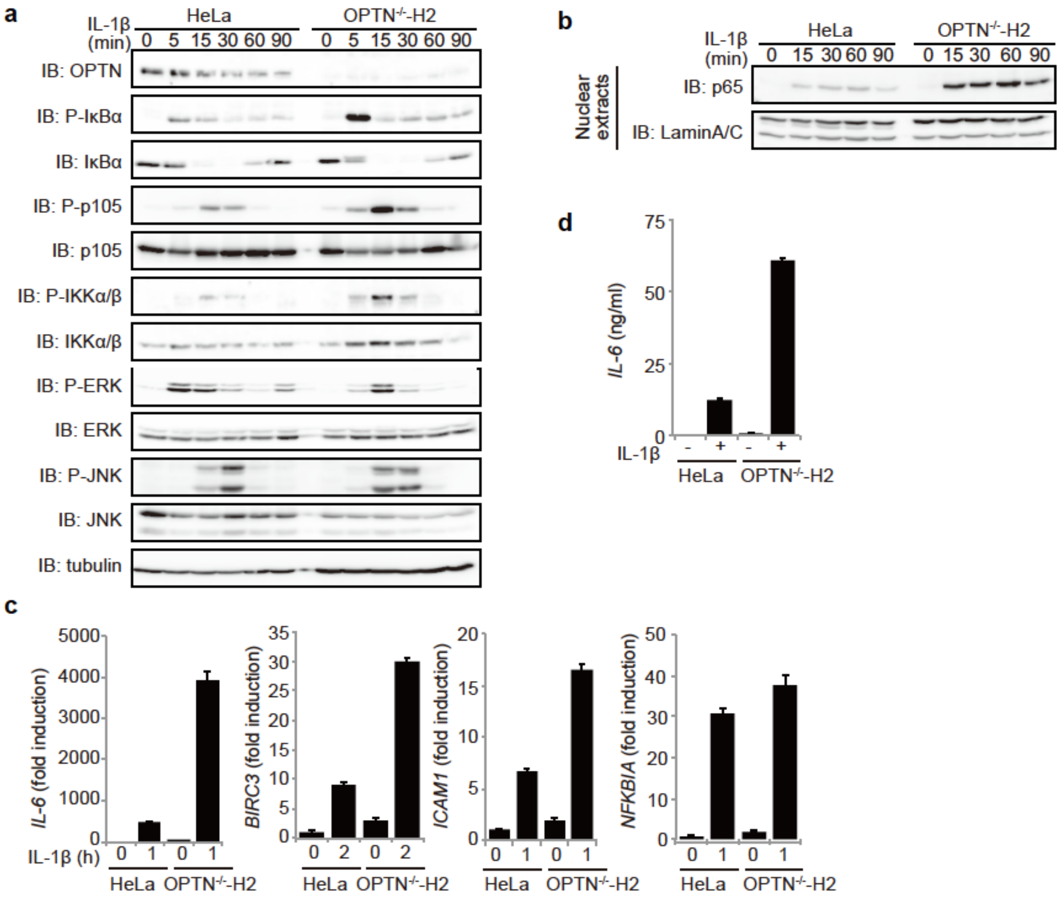

Supplementary Figure 8. *OPTN*-deletion causes enhanced IL-1β-induced NF-κB activation. (**a**) *OPTN*-KO and its parental HeLa cells were treated with IL-1β (1 ng ml^{-1}) for the indicated times, and cell lysates were immunoblotted with the indicated antibodies. (**b**) Enhanced intranuclear translocation of p65 in IL-1β-treated *OPTN*-KO cells. Nuclear extracts from IL-1β-treated *OPTN*-KO and parental HeLa cells were immunoblotted as in Fig. 3c. (**c**) qPCR analyses of NF-κB targets were performed, as shown in Fig. 3d. (**d**) Secretion of IL-6 from IL-1β-treated *OPTN*-KO and parental HeLa cells was measured by ELISA, as shown in Fig. 3e.

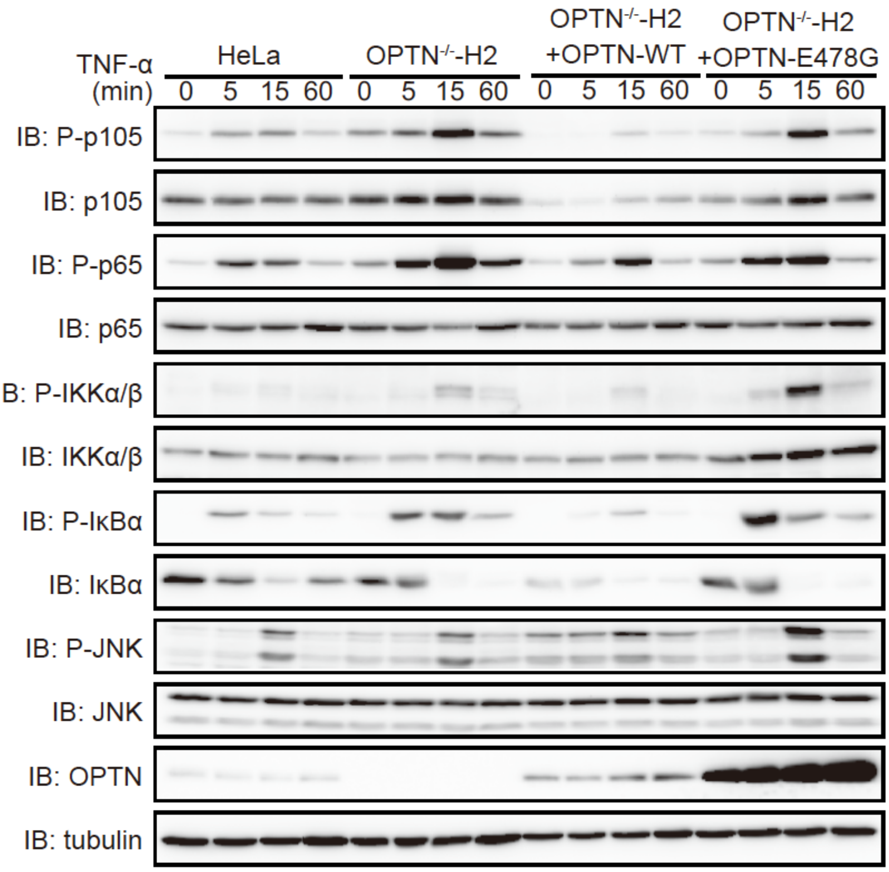

Supplementary Figure 9. Restoration of OPTN-WT, but not the E478G mutant, to *OPTN*-KO HeLa cells suppressed NF-κB activation. Stable cells expressing OPTN-WT or E478G mutant in *OPTN*-KO HeLa cells were constructed, and similar analyses to those shown in Fig. 3b were performed.

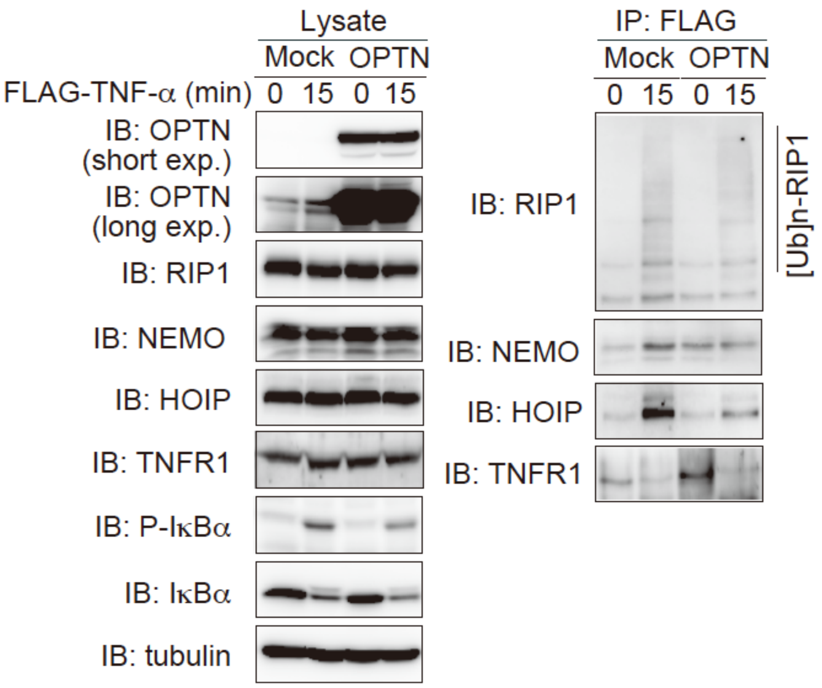

Supplementary Figure 10. Overexpression of OPTN suppresses TNFR signalling complex I formation. HeLa cells transiently overexpressing OPTN or mock plasmid were stimulated with FLAG-TNF-α, followed by immunoprecipitation by anti-FLAG beads. Cell lysates and immunoprecipitates were analyzed with the indicated antibodies.

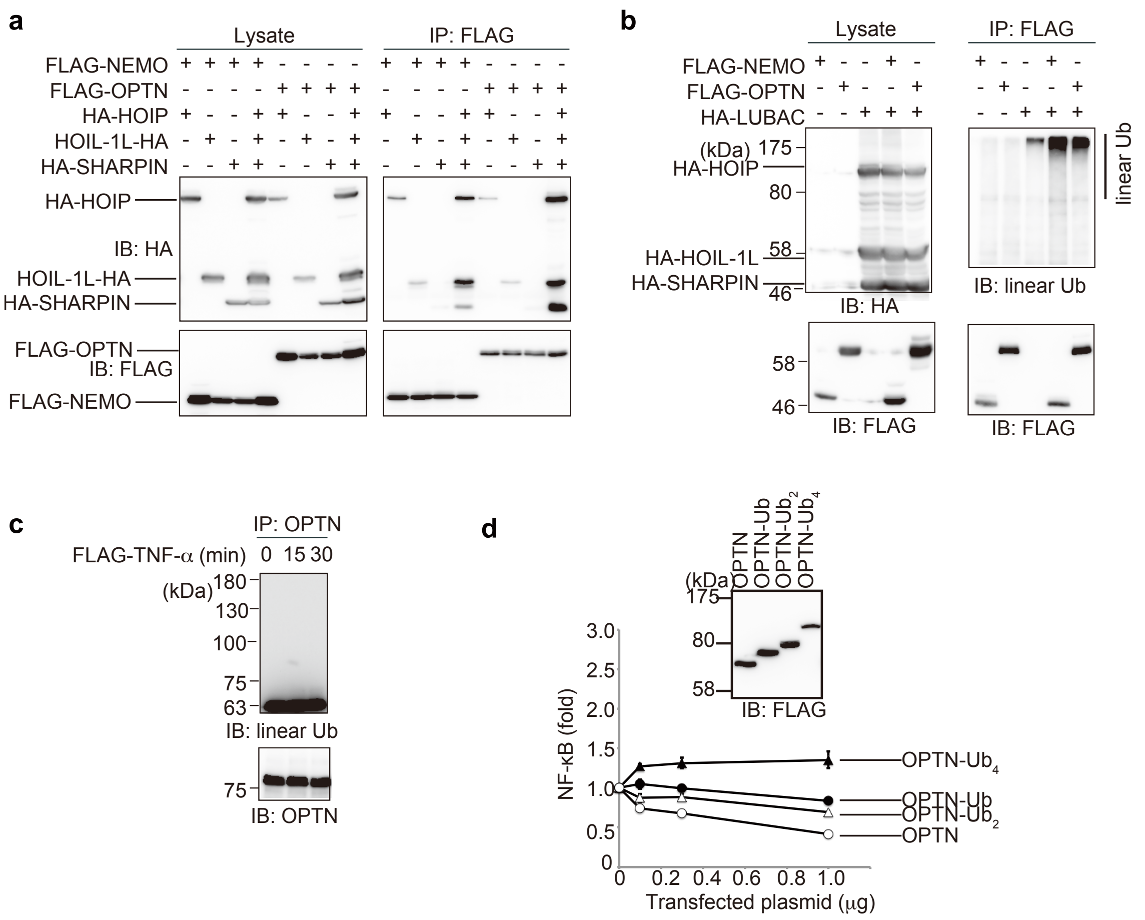

Supplementary Figure 11. Overexpressed OPTN is linearly ubiquitinated by LUBAC. (**a**) Association of OPTN and NEMO with LUBAC subunits of HOIP, HOIL-1L, and/or SHARPIN was examined by co-immunoprecipitation followed by immunoblotting analyses. (**b**) Linear ubiquitination of OPTN by LUBAC. NEMO or OPTN was coexpressed with LUBAC as indicated. Cells were lysed with 1% SDS and heated at 95°C. Cell lysates were immunoprecipitated with anti-FLAG beads, followed by immunoblotting with an anti-linear ubiquitin antibody. (**c**) Linear ubiquitination of endogenous OPTN was undetectable. HeLa cells were stimulated with FLAG-TNF-α for indicated times, and cell lysates (4.2 mg) were immunoprecipitated by anti-OPTN antibody, followed by

immunoblotted with the indicated antibodies. (**d**) OPTN fused with ubiquitin at the C-terminus did not potentiate NF-κB activation. Mono-, di- or tetra-ubiquitins were fused to the C-terminus of OPTN, and NF-κB luciferase reporter activity was examined with gradual increases in the transfected DNAs in HEK293T cells.

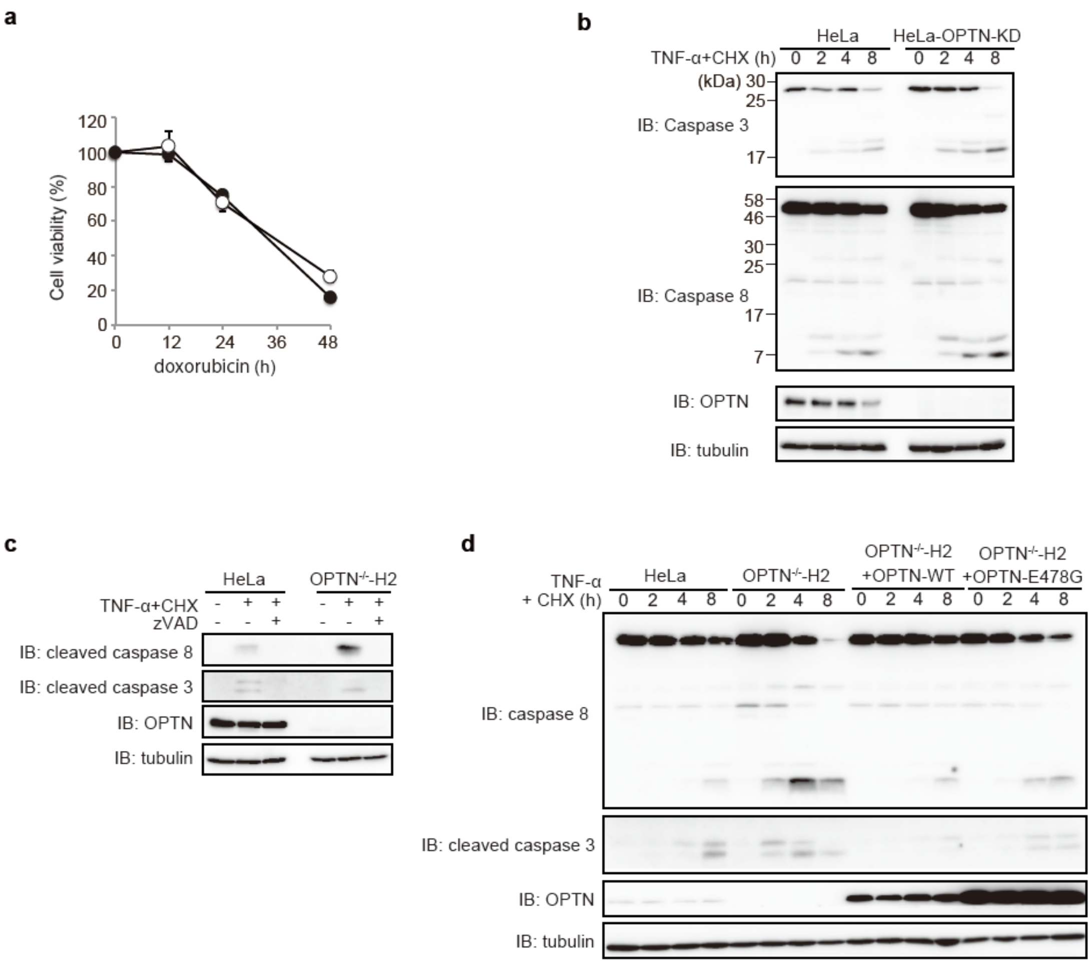

Supplementary Figure 12. Enhanced TNF-α-induced cell death in *OPTN*-KO and -KD cells. (**a**) Viability of *OPTN*-KO and its parental HeLa cells after doxorubicin-treatment showed no difference. Cell proliferation assays were performed as shown in Fig. 5a, after doxorubicin-treatment. (**b**) Accelerated activation of caspases in *OPTN*-KD cells. HeLa cells were transfected with control or *OPTN*-siRNA followed by treatment with TNF-α and CHX. Immunoblotting was performed using the indicated antibodies. (**c**) Caspase inhibitor suppressed TNF-α-induced caspase activation. *OPTN*-KO and its parental HeLa cells were treated with TNF-α+CHX and pan-caspase inhibitor

zVAD-FMK as indicated, and immunoblotting was performed using the indicated antibodies. (**d**) Restoration of OPTN-WT, but not the E478G mutant, to *OPTN*-KO HeLa cells suppressed TNF-α-induced apoptosis. Similar analyses to those shown in Fig. 5d were performed using OPTN-WT- and E478G mutant-restored cells.

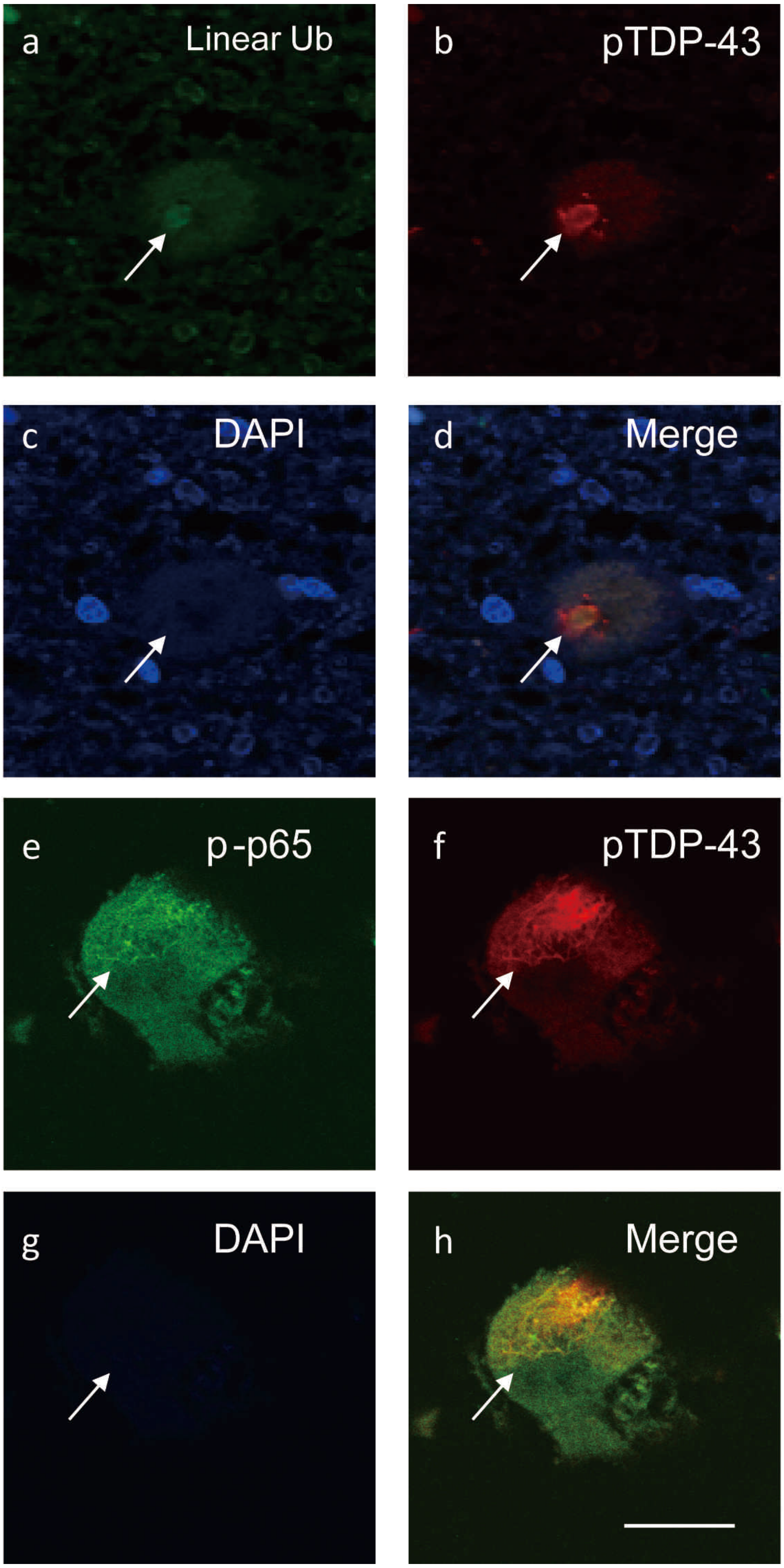
a
Linear Ub
b
pTDP-43
c
DAPI
d
Merge
e
p-p65
f
pTDP-43
g
DAPI
h
Merge

Supplementary Figure 13. Colocalization of P-TDP-43 and linear ubiquitin or P-p65. Immunofluorescent staining of linear ubiquitin and P-TDP-43 (**a-d**) or P-p65 and P-TDP-43 (**e-h**) was analyzed, using specimens from an OPTN-Q398X patient. Nuclear staining by DAPI and merged images are also shown. *Arrows* indicate immunoreactive inclusions. Bar: 25 μm.

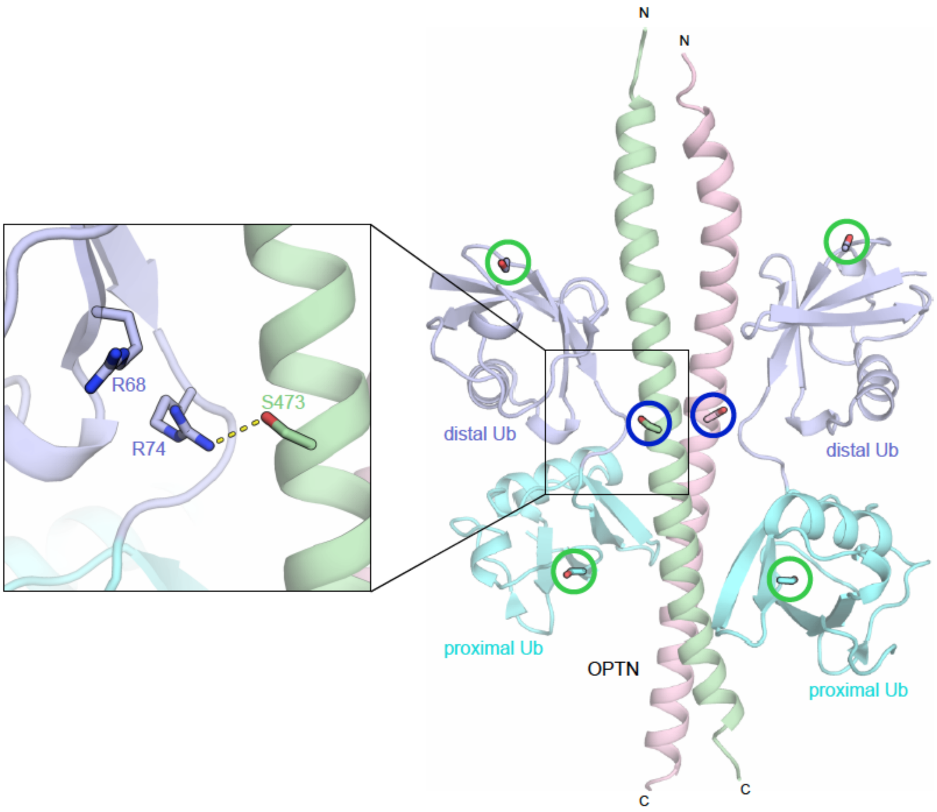

Supplementary Figure 14. Phosphorylation of S473 in OPTN, but not S65 in ubiquitin, may affect the linear ubiquitin-binding. Phosphorylation sites of S473 in OPTN and S65 in ubiquitin are circled in *blue* and *green*, respectively. Hydrogen bonding between S473 of OPTN and R74 of the distal ubiquitin is indicated in the *box*.

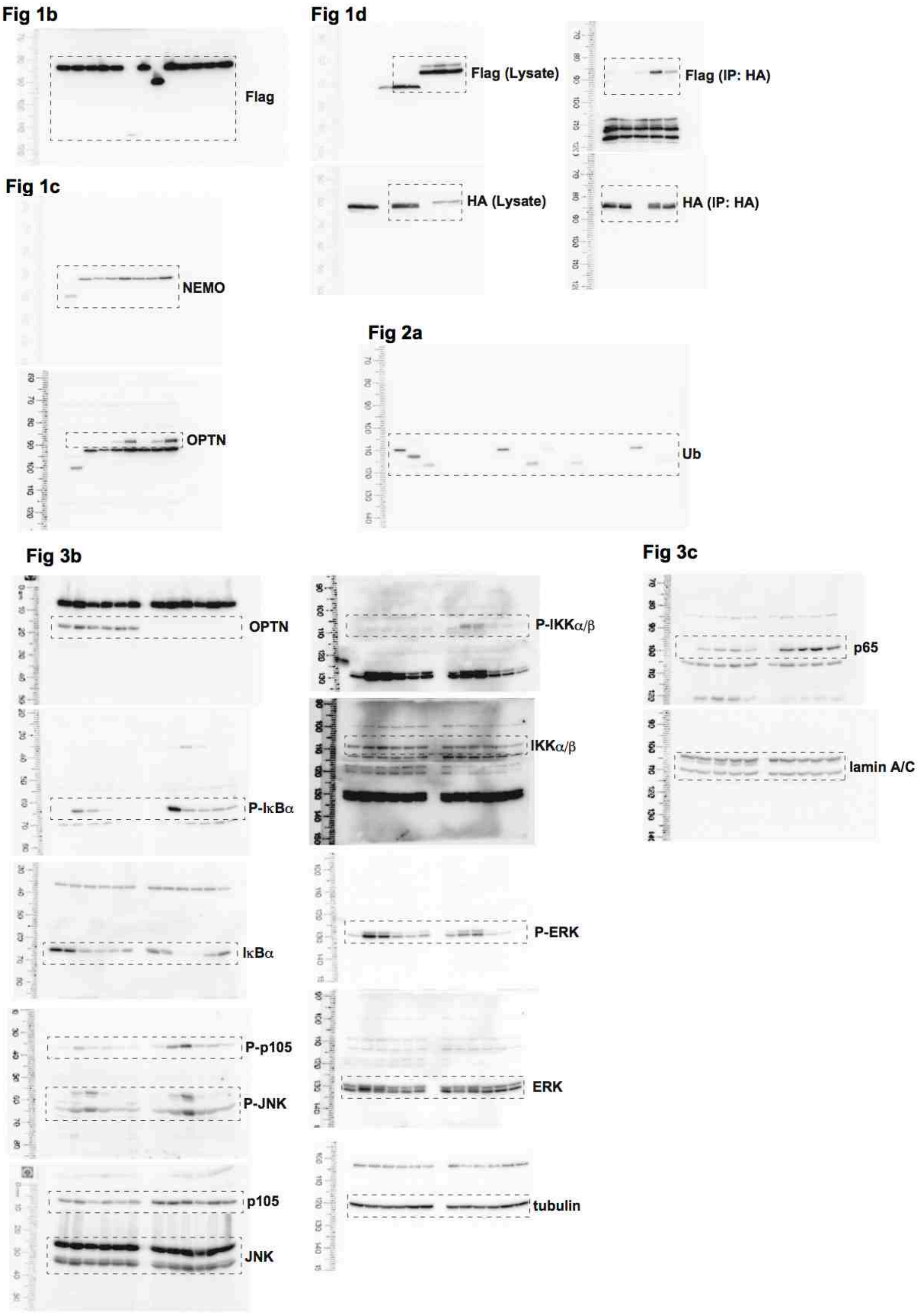

Supplementary Figure 15. Whole gel images of western blot results.

Fig 4a

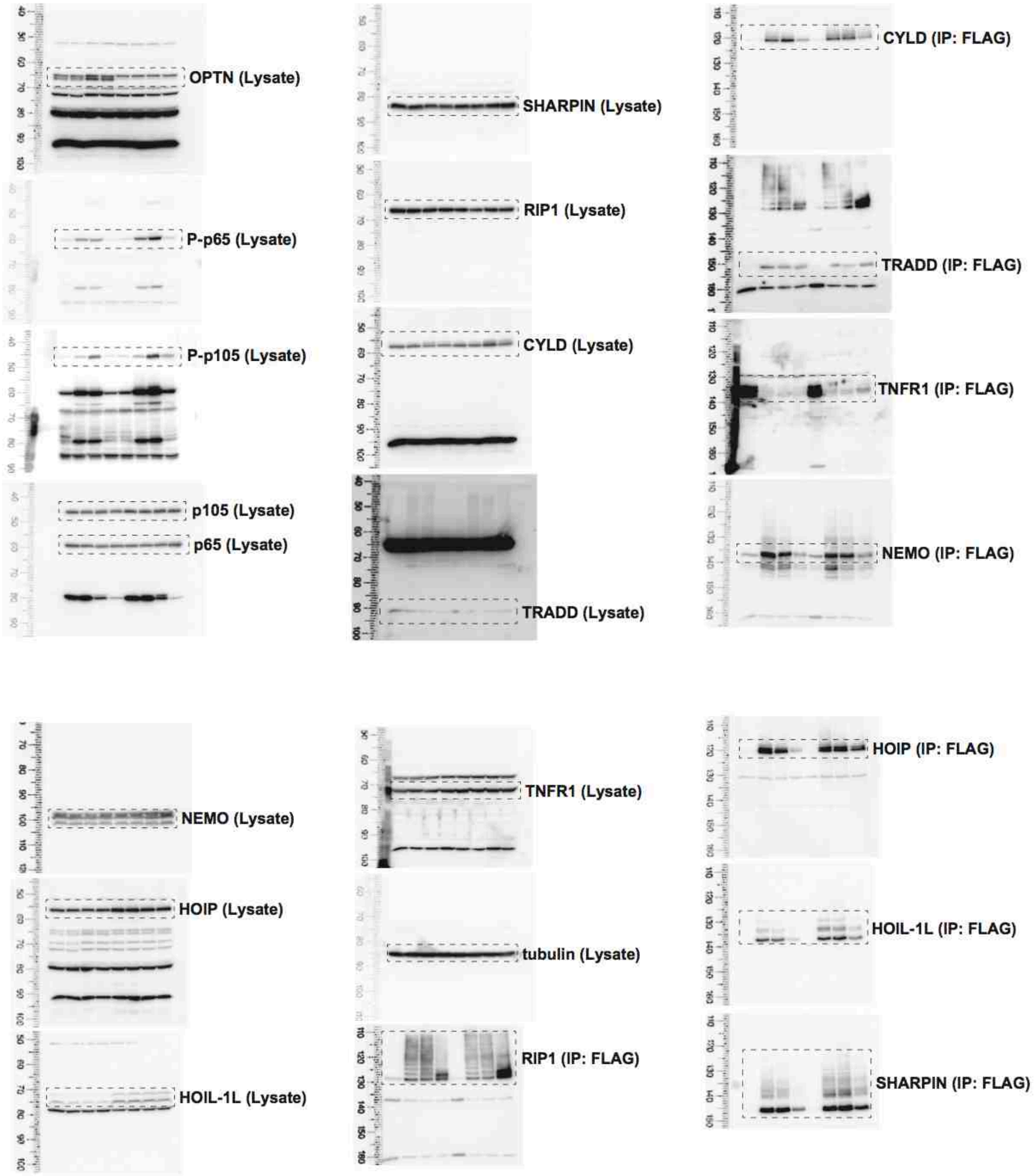

Fig 4b

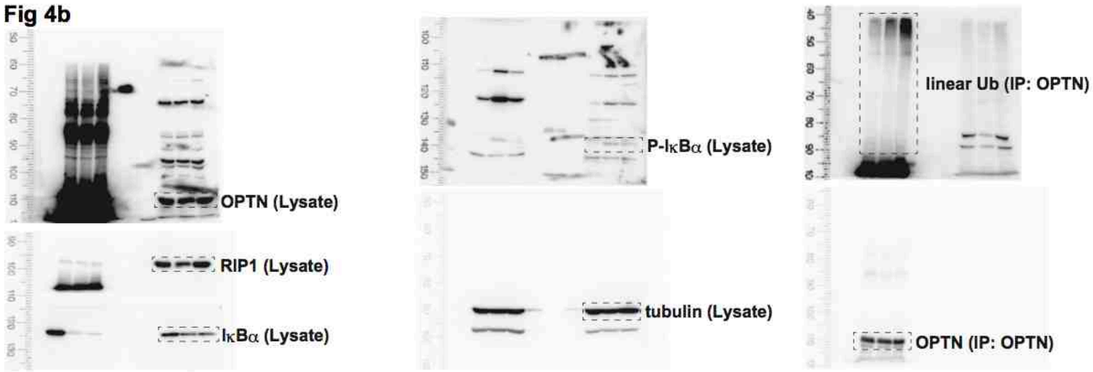

Supplementary Figure 15. cont.

Fig 4c

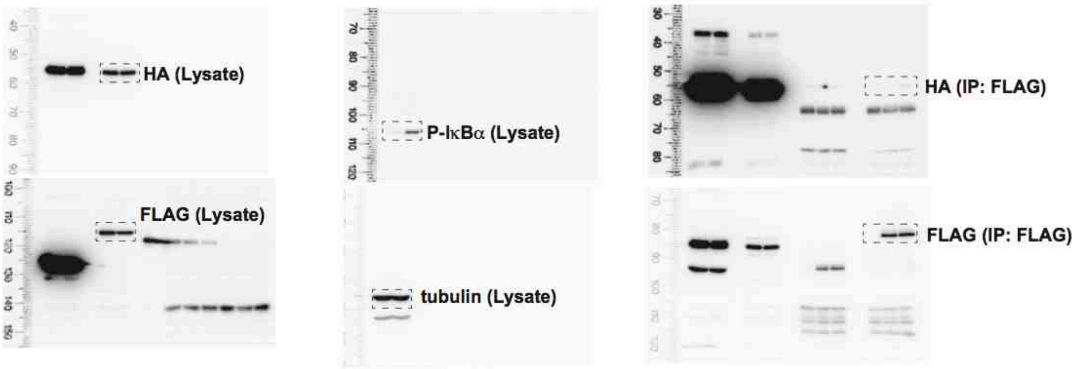

Fig 4d

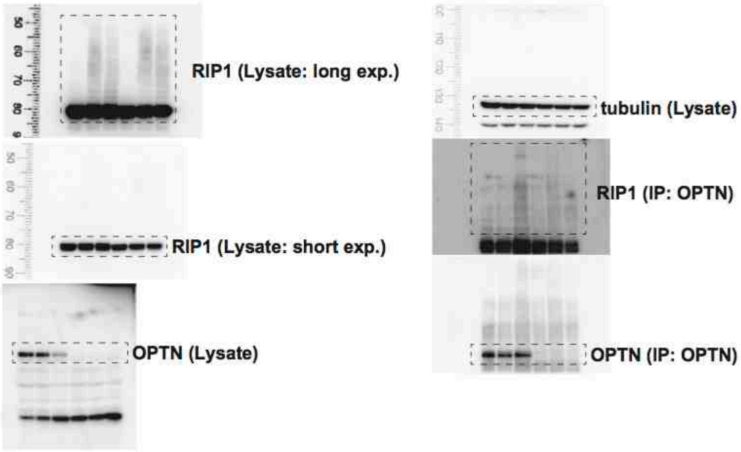

Fig 5d

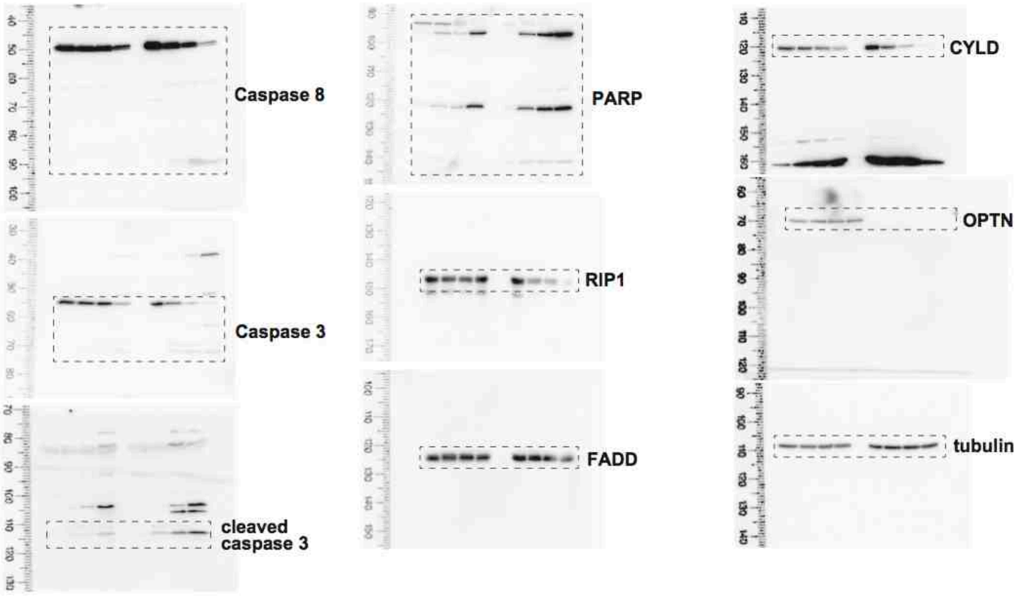

Supplementary Figure 15. cont.

Fig 5e

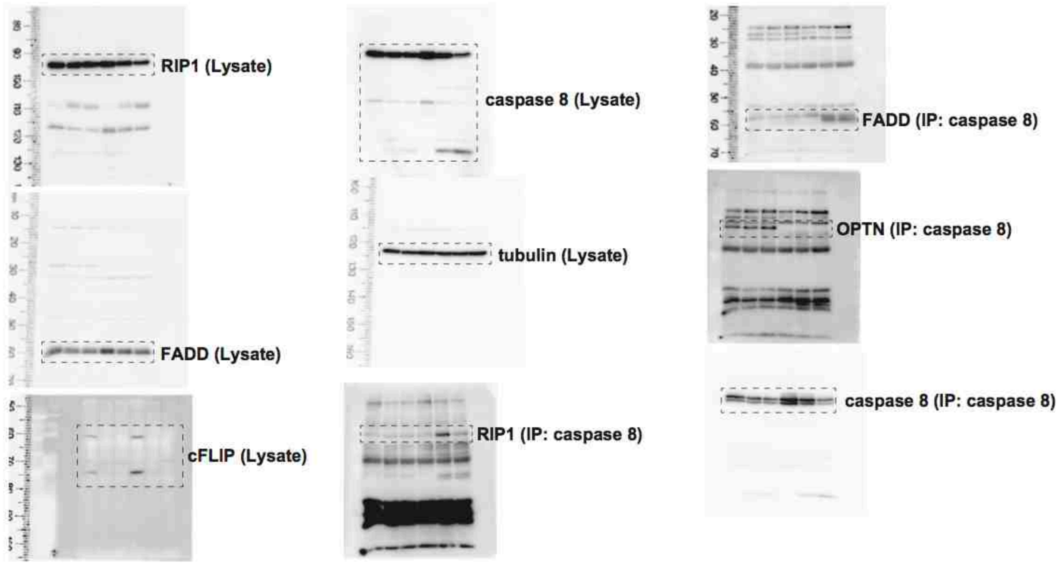

Fig 5f

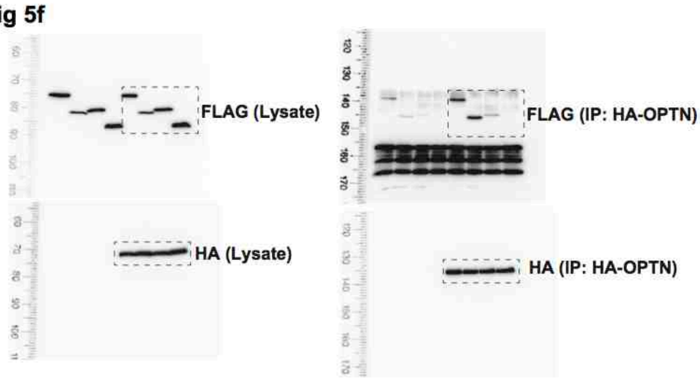

Fig 5g

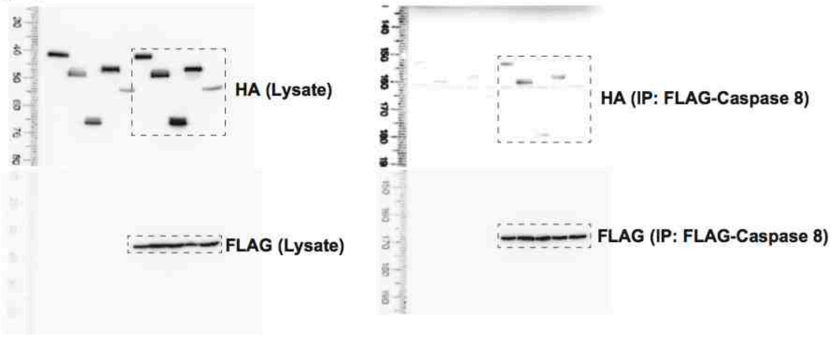

Supplementary Figure 15. cont.
